# Biocatalysis in Drug Design: Engineered Reductive Aminases (RedAms) Are Used to Access Chiral Building Blocks with Multiple Stereocenters

**DOI:** 10.1021/jacs.3c07010

**Published:** 2023-10-02

**Authors:** Arnau Rué Casamajo, Yuqi Yu, Christian Schnepel, Charlotte Morrill, Rhys Barker, Colin W. Levy, James Finnigan, Victor Spelling, Kristina Westerlund, Mark Petchey, Robert J. Sheppard, Richard J. Lewis, Francesco Falcioni, Martin A. Hayes, Nicholas J. Turner

**Affiliations:** † Department of Chemistry, University of Manchester, Manchester Institute of Biotechnology, 131 Princess Street, Manchester M1 7DN, United Kingdom; ‡ School of Engineering Sciences in Chemistry, Biotechnology and Health, Department of Industrial Biotechnology, KTH Royal Institute of Technology, AlbaNova University Center, 11421 Stockholm, Sweden; § Prozomix Ltd, Building 4, West End Ind. Estate, Haltwhistle NE49 9HA, United Kingdom; ∥ Early Chemical Development, Pharmaceutical Sciences, Biopharmaceuticals R&D, AstraZeneca, Mölndal, 431 50 Gothenburg, Sweden; ⊥ Medicinal Chemistry, Research and Early Development; Cardiovascular, Renal and Metabolism, Biopharmaceuticals R&D, AstraZeneca, Pepparedsleden 1, Mölndal, 431 50 Gothenburg Sweden; # Compound Synthesis and Management, Discovery Sciences, Biopharmaceuticals R&D, AstraZeneca, Mölndal, 431 50 Gothenburg, Sweden; ▽ Department of Medicinal Chemistry, Research and Early Development, Respiratory and Immunology (R&I), BioPharmaceuticals R&D, AstraZeneca, 43183 Mölndal, Sweden; ¶ Early Chemical Development, Pharmaceutical Sciences, Biopharmaceuticals R&D, AstraZeneca, CB21 6GP Cambridge, United Kingdom

## Abstract

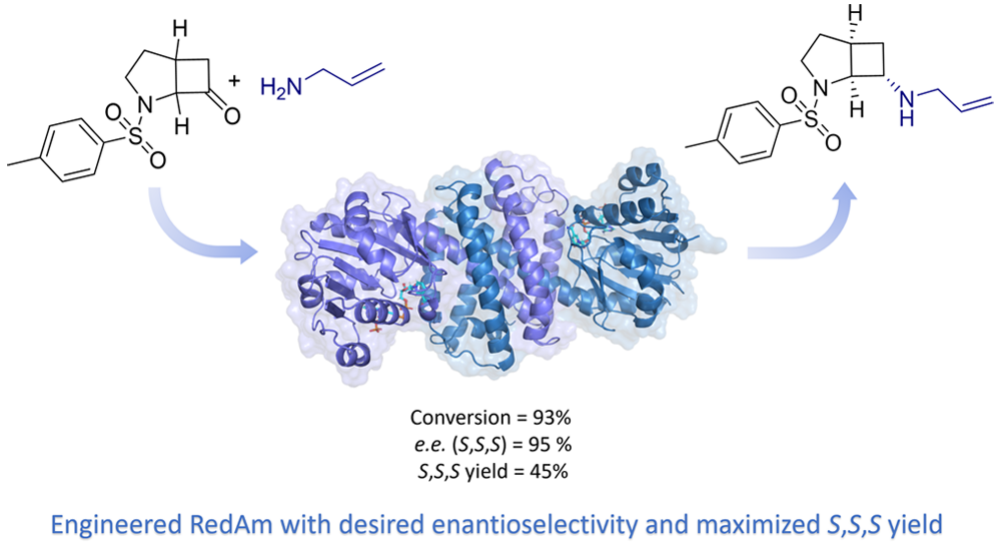

Novel building blocks are in constant demand during the search for innovative bioactive small molecule therapeutics by enabling the construction of structure–activity–property–toxicology relationships. Complex chiral molecules containing multiple stereocenters are an important component in compound library expansion but can be difficult to access by traditional organic synthesis. Herein, we report a biocatalytic process to access a specific diastereomer of a chiral amine building block used in drug discovery. A reductive aminase (RedAm) was engineered following a structure-guided mutagenesis strategy to produce the desired isomer. The engineered RedAm (IR-09 W204R) was able to generate the (*S*,*S*,*S*)-isomer **3** in 45% conversion and 95% ee from the racemic ketone **2**. Subsequent palladium-catalyzed deallylation of **3** yielded the target primary amine **4** in a 73% yield. This engineered biocatalyst was used at preparative scale and represents a potential starting point for further engineering and process development.

## Introduction

Chiral amines are common building blocks in the pharmaceutical industry that allow access to a plethora of biologically active compounds.^1−5^ They can be used for different applications, from development of small molecule pharmaceuticals^4−8^ to more complex therapeutics, such as proteolysis targeting chimeras (PROTACs),^9^ or for bioconjugation of peptides or proteins.^10−13^ The pharmaceutical industry is constantly looking for innovative ways to produce new bioactive compounds, which has increased the demand for novel building blocks in drug design.^14−20^ The wide structural diversity of such compounds facilitates the study of drug properties, such as permeability, potency, or secondary pharmacology. Current synthetic methodologies allow extensive coverage of available chemical space, but there are still opportunities for novel building blocks.^14,21−24^ Biocatalysis is a powerful synthetic methodology that can allow access to novel chemistries by exploiting mild reactions conditions, as well as the catalytic nature of the transformation, with high stereoselectivity driven by precise enzyme/substrate interactions.^17,18,25−27^

Importantly, biocatalytic synthesis can play a crucial role in drug discovery since enzymes are tunable catalysts that allow for excellent control of chemoselectivity, regioselectivity and enantioselectivity.^25,28,29^ Imine reductases (IREDs) are one emerging platform of biocatalyst^30−34^ that perform a variety of reactions, including cyclic imine reduction,^31,35,36^ reductive amination (RedAms),^34,37,38^ and alkene reduction (EneIRED).^3,39^ Interestingly, individual IREDs can behave simultaneously as an IRED, a RedAm, or an EneIRED depending on the substrate used while still maintaining excellent chemoselectivity.^3^

## Results and Discussion

**Figure 1:**
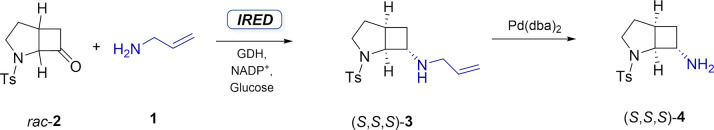
Combined biocatalytic resolution and reductive amination of racemic ketone **2** with allylamine **1** forming (*S,S,S*)-3, which is a key intermediate for the desired primary amine (*S,S,S*)-**4**.

Herein, we address the enzymatic synthesis of a chiral building block (*S,S,S*)-**4**, which was required for a medicinal chemistry discovery program and proved to be particularly challenging to obtain by organic synthesis. We initially envisioned directly accessing this primary chiral amine via a RedAm-mediated reductive amination with ammonia. However, although RedAms have been previously shown to use ammonia as the nucleophile,^40^ they were found to be inactive with racemic ketone **2**. We therefore elected to use allylamine **1**, which typically shows high activity with a wide range of different RedAms and should generate a product that could be readily converted to primary amine **4** (Figure 1).

A metagenomic panel of IREDs and RedAms,^36^ containing different biocatalysts, was screened to identify active enzymes capable of converting the substrate ketone **2** to the product **3**. A previously reported colorimetric high-throughput screening method (HTS),^36^ which operates in the oxidative direction and uses the product of the reaction **3** to identify active enzymes, was initially employed to select enzymes for further characterization.

Any hits obtained from this primary screen were subsequently confirmed by examining the reductive amination of **2** with allylamine **1** in the synthetic direction. In addition, on the basis of previous experience, several other IREDs were selected because of known substrate promiscuity. Two of these IREDs were found to be active; IR-09 and IR-20 exhibited >95% and 47% conversion, respectively.

All biotransformations were performed with glucose and glucose dehydrogenase (GDH) as the NADPH recycling system. For all reactions, formation of alcohol was observed to some extent, likely because of either ketone reduction catalyzed by GDH^41,42^ and/or other ketoreductases (KREDs) present in the cell-free extract (CFE).^43,44^ Consequently, IR-09 was purified, and reactions were performed with pure IR-09 in the absence of GDH; under these conditions, no alcohol formation was observed, thereby confirming the excellent chemoselectivity of IR-09. (Supplementary Figure S8).

**Figure 2:**
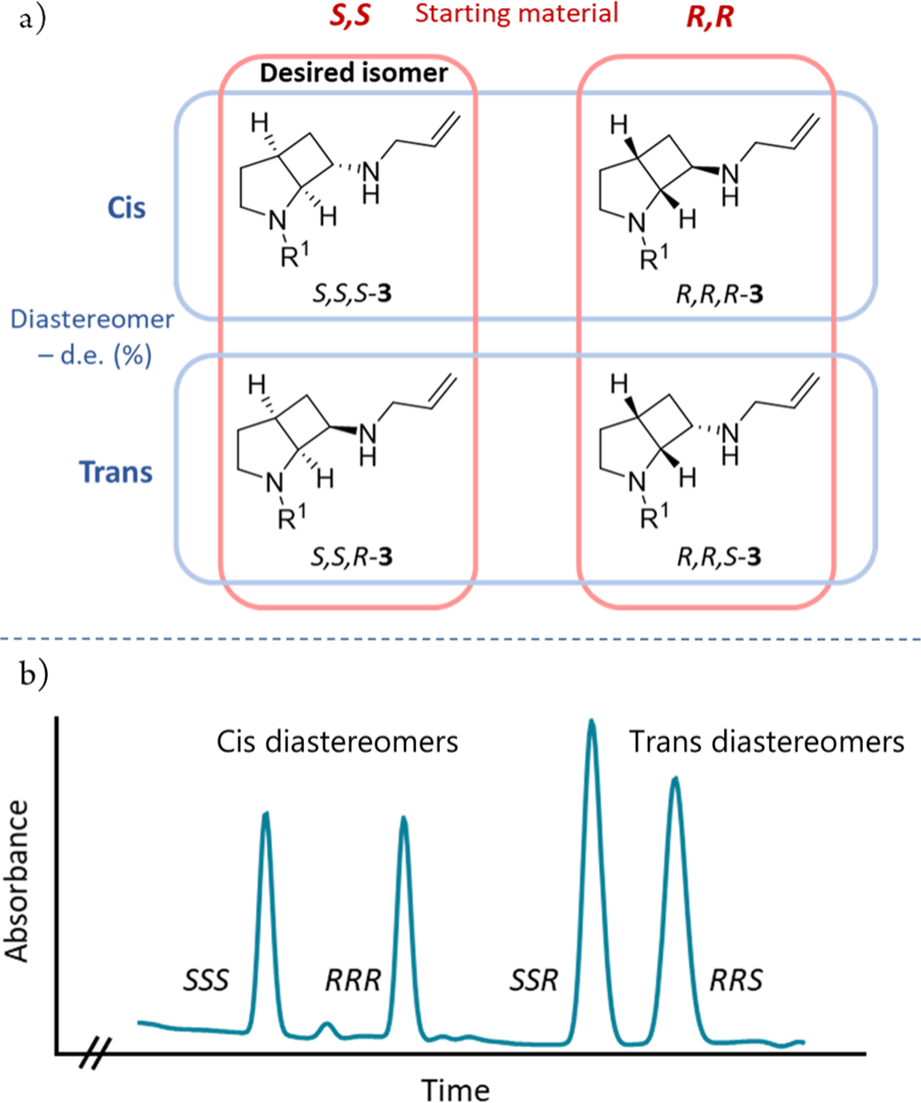
(a) The four possible reductive amination products (*S,S,S*)-**3**, (*R,R,R*)-**3**, (*S,S,R*)-**3**, and (*R,R,S*)-**3** derived from ketone (±)-**2**; (b) SFC chromatogram of the stereoisomers.

Unfortunately, none of the active enzymes generated the desired diastereomer (*S,S,S*)-**3** (Table 1). In fact, all of the hits preferentially yielded the trans-diastereomers (*S,S,R*)-**3** and (*R,R,S*)-**3** as the major products, as shown by chiral supercritical fluid chromatography (SFC) (Figure 2). For example, IR-09 gave 96% conversion to predominantly a mixture of (*S,S,R*)-**3** and (*R,R,S*)-**3** (expressed as 85% de trans) with a small amount of (*R,R,R*)-**3** of high ee (99%). A phylogenetic tree was constructed to identify further homologues of IR-09 and IR-20 that might possess similar levels of activity but with altered diastereoselectivity. In total, eight homologues of IR-09 were screened and showed some activity, but none exhibited the desired diastereoselectivity.

**Table 1:** Active Enzymes for the Reductive Amination of (±)-**2** with Allylamine **1**^a^

IRED	conversion (%)	de trans (%)	ee (*R,R,R*) (%)
IR-09	96	85	99
IR-16	89	93	99
IR-20	47	34	99
IR-61	53	99	
IR-202	72	92	99
IR-361	29	99	

a All enzymes yielded (*S,S,R*)-**3** and (*R,R,S*)-**3** as the major diastereomers. Reaction conditions: 10 mM rac-**2**, 10 amine equiv of **1**, 4 mg mL^–1^ of imine reductase cell-free extract (IRED CFE), 0.5 mg mL^–1^ of glucose dehydrogenase (GDH), 40 mM glucose, 5% v/v of DMSO, 100 mM Tris buffer pH 8. See the Supporting Information Section 4 for equations details.

In order to gain access to the desired diastereomer, we therefore considered engineering one of our active hits. It was decided to initially explore a structure-guided approach in order to alter the stereoselectivity of IR-09.

Clearly, the major challenge for protein engineering of IR-09 was to generate variants with both high kinetic selectivity for the ketone enantiomer [i.e., preference for reaction of (*S,S*)-**2** over (*R,R*)-**2**], as well as high stereoselectivity for the reductive amination step [i.e., preference for formation of (*S,S,S*)-**2** over (*S,S,R*)-**2**] (Figure 2a). Encouragingly, previous studies have shown that RedAms can be engineered for high kinetic selectivity for racemic amines.^5^

Since IR-09 exhibited higher activity than the other enzyme hits, this enzyme was selected as the backbone for engineering. A structure-guided mutagenesis approach was developed around the principle that the diastereoselectivity and enantioselectivity of the enzyme would be based on the orientation of the substrate in the active site. A crystal structure of IR-09 was available and used to identify activity site residues that could potentially play a key role in determining the stereoselectivity of the reaction.

**Figure 3:**
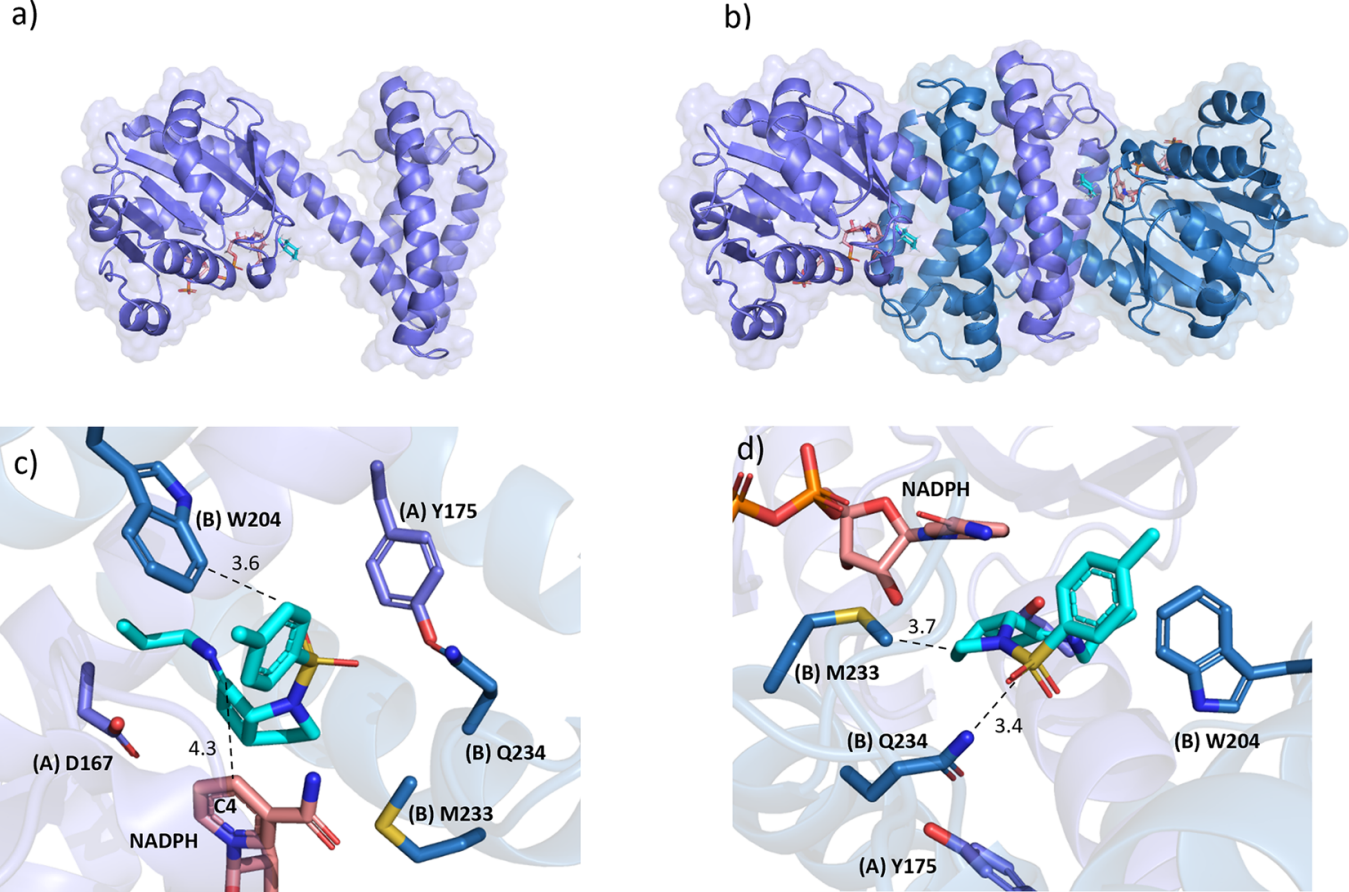
(a) Crystal structure of IRED-09 (purple) in complex with NADPH (pink) and *N*-cyclopropylcyclohexanamine; there is only one molecule in the asymmetric unit. (b) IR-09 biological dimer. (c) Active site of IR-09 with the imine intermediate of **3** modeled into the active site showing distances (Å) from C4 of the nicotinamide ring of NAPDH and (B) W204 to the electrophilic carbon of the ligand. (d) The view is rotated 180 deg to observe the active site from the opposite perspective and show distances from (B) M233 and (B) Q234 to the ligand.

The crystal structure of IR-09 contained a monomer in the asymmetric unit with the biological dimer being generated through crystallographic symmetry (see the Supporting Information Section 9 for details.). The crystal structure was obtained as a ternary complex with NADPH and *N*-cyclopropylcyclohexanamine. *N*-Cyclopropylcyclohexanamine is a considerably smaller ligand than **3**, thus AutoDock Vina^45^ flexible docking was performed to find a suitable pose for **3**. Amino acid residues within 5 Å of the substrate were selected and allowed to freely rotate. Thereafter, the active site containing the imine intermediate was studied, and residues having a potential influence on the orientation of the ligand were identified (Figure 3). W204 was found at a distance of 3.6 Å from the ligand, and a similar situation was observed for M233 and Q234, which were at 3.7 and 3.4 Å, respectively. These observations were consistent with previous experience of corresponding residues in different RedAms.^37^ A multiple sequence alignment was performed and used to confirm that these three residues were conserved across all hits and in AspRedAm.

As a result of these docking studies, amino acid residues W204, M233, and Q234 were targeted for site-directed mutagenesis (SDM) with either alanine or serine as the initial replacements. W204A and W204S were identified as the most promising variants (Table S2 and Supplementary Figures S10 and S11) since they both gave rise to a partial increase in diastereoselectivity for the cis diastereomer and, most importantly, they both generated the previously unobserved (*S,S,S*)-enantiomer. Assignment of the absolute configuration of all four diastereomers of **3** was established by visible circular dichroism (VCD) (see the Supporting Information Section 15). Variants M233A and Q234A also exhibited a change in diastereoselectivity, but both of these enzymes continued to favor the formation of the undesired trans diastereomers.

**Figure 4:**
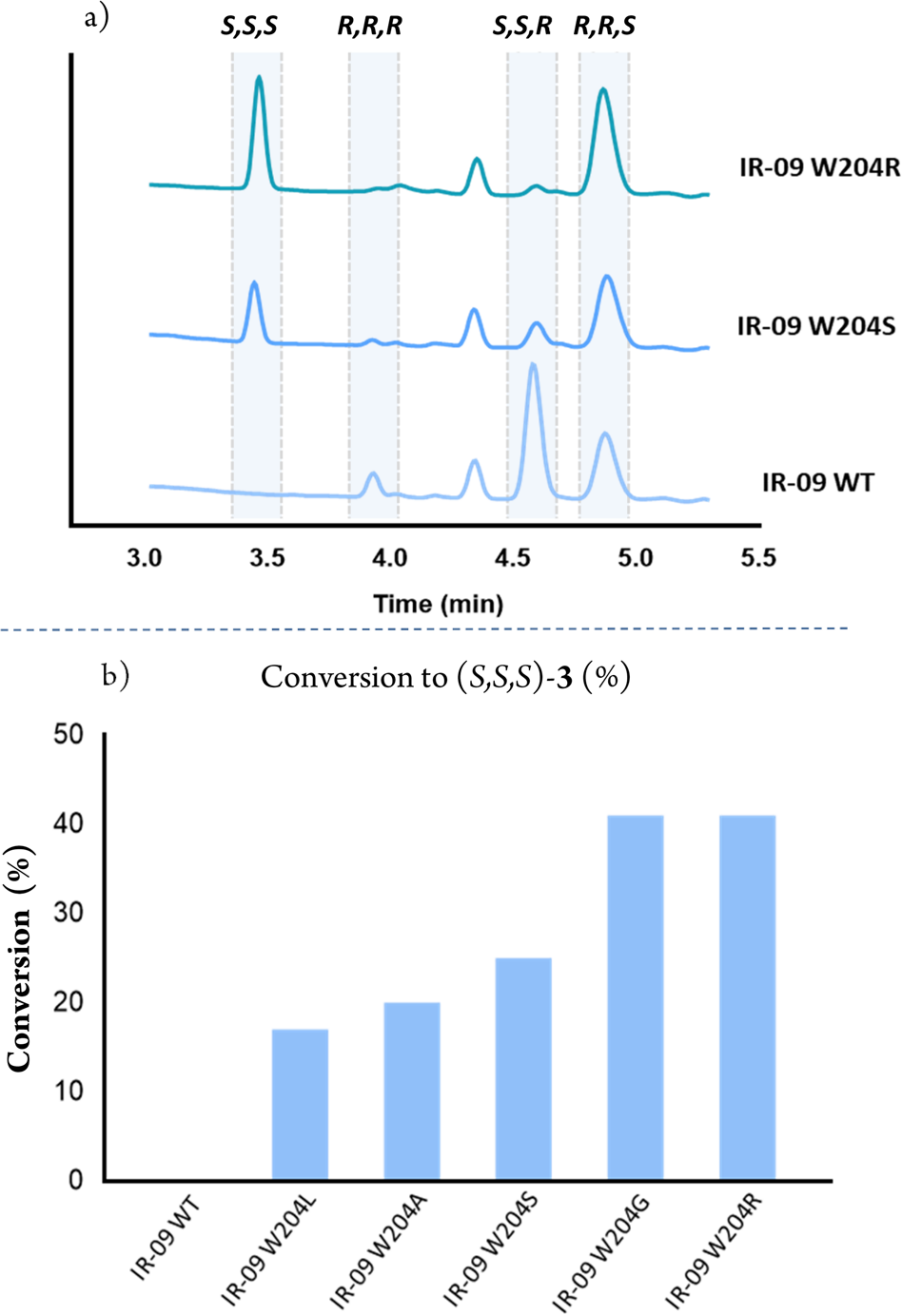
(a) SFC chromatograms comparing the isomer production of IR-09 WT, the best SDM variant, and the best SSM variant (peak at rt = 4.4. min corresponds to the alcohol). (b) Comparison between WT and the best variants for the production of (*S,S,S*)-**3**. Conversion to *(S,S,S*)-**3** (%) = (*S,S,S*) yield × conversion.

Since both IR-09 W204 variants still generated considerable amounts of the (*S,S,R*)-enantiomer (Figure 4a), site saturation mutagenesis (SSM) was performed in order to obtain a variant with higher selectivity. Three new variants were identified that generated the (*S,S,S*)-enantiomer, namely W204L, W204R, and W204G. Among these variants, IR-09 W204R exhibited the highest selectivity for the (*S,S,S*)-enantiomer, with 45% *S*,*S*,*S* yield. In contrast, W204L resulted in a decrease in enantioselectivity to lower levels than those of either W204A or W204S.

The relative activity of these new variants was also assessed. Previously, all reactions were performed with a large excess of amine (10 equiv). Screening at a lower amine concentration (1 equiv) revealed that IR-09 WT, IR-09 W204S, and IR-09 W204R all exhibited good levels of activity, especially W204R, which yielded 60% conversion (Supplementary Figure S1). Most variants and WT enzymes exhibited similar degrees of enantioselectivity toward the (*R,R*)- and (*S,S*)-**2** ketone, since the differences between the amount of (*S,S,S*) and (*S,S,R*) in comparison with the amount of (*R,R,R*) and (*R,R,S*) formed was always below 2%.

IR-09 W204G is clearly selective for the (*S,S*)-ketone and was the first variant identified to achieve selective reductive amination while simultaneously performing a kinetic resolution of the starting material. Variant W204G yielded an (*S,S,S*) yield of 56% with 75% conversion (Table 2 and Supplementary Figure S16).

**Table 2:** Characterization of WT and IR-09 Variants with Respect to Both the Diastereoselectivity and Enantioselectivity^a^

IR-09	conversion (%)	de cis (%)	ee (*S,S,S*) (%)	*S,S,S* yield (%)
WT	96	–85		0
W204L	89	–47	85	19
W204A	92	–48	64	21
W204S	93	–39	80	28
W204G	75	15	96	56
W204R	93	–9	95	45

a Reaction conditions: 10 mM rac-**2**, 10 amine equiv of **1**, 4 mg mL^–1^ of IRED CFE, 0.5 mg mL^–1^ of GDH, 40 mM glucose, 5% v/v of DMSO, 100 mM Tris buffer pH 8. See the Supporting Information Section 4 for equation details.

Finally, preparative biotransformations were carried out to assess the scalability of the reductive amination process. IR-09 W204R was selected for a 50 mL scale reaction, which was carried out on an EasyMax system (Mettler Toledo). The reactions resulted in 91% conversion and yielded the same distribution of stereoisomers as the analytical scale reactions [ee of (*S,S,S*)-**3** = 95%]. The product mixture was extracted with methyl *tert*-butyl ether (MTBE) and subsequently purified by preparative supercritical fluid chromatography (SFC) to yield pure stereoisomers of amine **3**, which were characterized by NMR and mass spectrometry (Supplementary Section 15). Finally, deallylation of (*S,S,S*)-**3** was performed with Pd(dba)_2_ and DPPB in THF for 2 h followed by extraction with EtOAc to yield the target *N*-tosyl-protected amine product (*S,S,S*)-**4** in 73% yield as a colorless oil (Supporting Information Sections 13 and 16).

## Conclusions

In summary, we have demonstrated that biocatalysis is a powerful tool to enable the production of difficult to access complex chiral amine building blocks for drug design. In this context, structure-guided mutagenesis proved to be a rapid way of tuning the selectivity of the wild-type biocatalyst for the synthesis of a target molecule with multiple stereocenters. Both the stereoselectivity and stereospecificity of the enzyme were further improved by saturating a key active site residue, which enabled reductive amination with concomitant kinetic resolution. The engineered enzyme retained high levels of conversion and selectivity on a preparative scale, thereby showing the potential for further evolution for early chemical development.

## Supporting Information Available

Experimental section, including general information, experimental procedures, enzyme and primers sequences, chromatograms of biotransformations, and characterization of compounds (PDF)
