# Supplementary material for: Biocatalysis in Drug Design: Engineered Reductive Aminases (RedAms) Are Used to Access Chiral Building Blocks with Multiple Stereocenters
Source: J Am Chem Soc. 2023 Oct 2;145(40):22041–6. doi: 10.1021/jacs.3c07010 (PMC10571080; doi:10.1021/jacs.3c07010)
Supplement: Supplementary file 1 — ja3c07010_si_001.pdf [file ja3c07010_si_001.pdf]

Supplementary Information for

Biocatalysis in drug design: Engineered reductive aminases (RedAms) to access chiral building blocks with multiple stereocenters.

Arnau Rué Casamajo[§], Yuqi Yu[§], Christian Schnepel[∮], Charlotte Morrill[§], Rhys Barker[§], Colin W. Levy[§], James Finnigan[ᵍ], Victor Spelling[ᵗ], Kristina Westerlund[ᵻ], Mark Petchey[ᵹ], Robert J. Sheppard[ᵻ], Richard J. Lewis[ϴ], Francesco Falcioni[Ɣ], Martin A. Hayes[ᵹ], Nicholas J. Turner[§]*

[§]Department of Chemistry, University of Manchester, Manchester Institute of Biotechnology, 131 Princess Street, Manchester M1 7DN, United Kingdom

[∮]School of Engineering Sciences in Chemistry, Biotechnology and Health, Department of Industrial Biotechnology, KTH Royal Institute of Technology, AlbaNova University Center, 11421 Stockholm, Sweden

[ᵍ]Prozomix Ltd, Building 4, West End Ind. Estate, Haltwhistle NE49 9HA, United Kingdom

[ᵗ]Early Chemical Development, Pharmaceutical Sciences, Biopharmaceuticals R&D, AstraZeneca, 431 50 Mölndal, Gothenburg, Sweden

[ᵻ]Medicinal Chemistry, Research and Early Development; Cardiovascular, Renal and Metabolism, Biopharmaceuticals R&D, AstraZeneca, Pepparedsleden 1, Mölndal, 431 50 Gothenburg, Sweden

[ᵹ]Compound Synthesis and Management, Discovery Sciences, Biopharmaceuticals R&D, AstraZeneca, 431 50 Mölndal, Gothenburg, Sweden

[ϴ]Department of Medicinal Chemistry, Research and Early Development, Respiratory and Immunology (R&I), BioPharmaceuticals R&D, AstraZeneca, 43183 Mölndal, Sweden

[Ɣ]Early Chemical Development, Pharmaceutical Sciences, Biopharmaceuticals R&D, AstraZeneca, CB21 6GP, Cambridge, United Kingdom

Biocatalysis, drug discovery, RedAms, reductive amination, pharmaceutical building blocks, diastereoselectivity, semi rational mutagenesis.

Corresponding author email: Nicholas.Turner@manchester.ac.uk

Table of Contents

1. Material and Equipment 1
   1.1. Chemicals and equipment 1
   1.2. Supercritical Fluid Chromatography (SFC) analysis 1
   1.3. Preparative Supercritical Fluid Chromatography analysis 2
   1.4. Vibrational circular dichroism (VCD) analysis 2
2. Bicyclic ketone synthesis 2
3. Diastereomers and enantiomers 4
4. Formulas and equations 4
5. Biocatalyst gene and protein sequence 5
6. Procedure for the production of biocatalysts 6
   6.1. Origin and cloning 6
   6.2. Expression 6
   6.3. Cell-free enzyme (CFE) preparation 7
   6.4. Biocatalyst purification 7
7. General procedure for biotransformations 7
   7.1. Amine equivalences optimization 8
   7.2. IR-09 close homologues 9
8. Primers sequences 9
9. Crystallization IR-09 10
10. Docking 11
11. Procedure for mutagenesis 12
   11.1. Site directed mutagenesis (SDM) 12
   11.2. Site saturation mutagenesis (SSM) 13
12. Preparative-scale reaction 13
13. Procedure for deallylation 14
14. Chromatograms and MS spectra 14
   14.1. Analytical SFC chromatograms 14
      14.1.1. Wild Type enzymes 14
      14.1.2. Pure IR-09 14
      14.1.3. Site directed mutagenesis (SDM) variants 16
      14.1.4. Site saturated mutagenesis (SSM) variants 17
   14.2. Preparative SFC chromatogram and MS spectrums 18

15. Vibrational circular dichroism (VCD) results 21
15.1. Experimental infra-red and VCD spectra 21
15.2. Computational Spectral Simulations 22
15.3. Fit between calculated and experimental IR and VCD spectra 25
16. NMR analysis 27
16.1. Bicyclic ketone synthesis 27
16.1.1. *N*-(But-3-en-1-yl)-4-methylbenzenesulfonamide 27
16.1.2. *N*-(But-3-en-1-yl)-4-methyl-*N*-(2-oxo-2-(pyrrolidin-1-yl)ethyl)benzenesulfonamide 28
16.1.3. *rac*-(1*S*,5*S*)-2-Tosyl-2-azabicyclo[3.2.0]heptan-7-one (2) 29
16.2. Isolated products spectra 30
16.2.1. (*S*,*S*,*S*)-3 30
16.2.2. (*R*,*R*,*R*)-3 31
16.2.3. (*S*,*S*,*R*)-3 32
16.2.4. (*R*,*R*,*S*)-3 33
16.2.5. Deallylated 3 product 34
17. References 35

1. Material and Equipment

1.1. Chemicals and equipment

Commercially available chemicals and solvents were used without further purification and were purchased from Fluorochem (Hadfield, Derbyshire, UK), Alfa Aesar (Karlsruhe, Germany), Prozomix (Haltwhistle, Northumberland, UK) or Sigma-Aldrich (Poole, Dorset, UK) and stated otherwise. Racemic starting material was provided by AstraZeneca.

Preparative-scale reactions were performed on a Mettler Toledo EasyMax 102 with temperature, pH and stirring control.

Cells were sonicated with a 4-probe Qsonica sonicator and cell-free extract was lyophilized with a Buchi Lyovapor L-200 at 0.05 mbar.

1.2. Supercritical Fluid Chromatography (SFC) analysis

Supercritical fluid chromatography analysis was performed using an Agilent 1290 Infinity II with a SFC control module equipped with a Daicel ChiralPack IG-3 column (3 µm, 3mm and 50mm). Different methods were to analyse biotransformations:

7.5 minutes method (Original method):

Eluent CO_2/MeOH + 0.1% NH_3 starting for 2 minutes at 95% CO_2, a gradient for 3 minutes up to 40% MeOH + 0.1% NH_3, isocratic during 1 minutes, 0.1 minute up down to 5% MeOH + 0.1% NH_3, and equilibration at 5% for 1.4 minutes. Flow: 1 ml/min; column temperature 40 °C; injection volume 3 or 4 µl; UV detection at λ = 210 nm and λ = 234 nm.

6 minutes method (Corroboration method for best SSM variants):

Eluent CO_2/MeOH + 0.1% NH_3 starting for 1 minute at 95% CO_2, a gradient for 2.5 minutes up to 40% MeOH + 0.1% NH_3, isocratic during 1 minutes, 0.1 minute up down to 5% MeOH + 0.1% NH_3, and equilibration at 5% for 1.4 minutes. Flow: 1 ml/min; column temperature 40 °C; injection volume 3 or 4 µl; UV detection at λ = 210 nm and λ = 234 nm.

4 minutes method (Method for SSM plate):

Eluent CO_2/MeOH + 0.1% NH_3 starting with a gradient for 2 minutes up to 50% MeOH + 0.1% NH_3, isocratic during 0.5 minutes, 0.1 minute up down to 5% MeOH + 0.1% NH_3, and equilibration at 5% for 1.4 minutes. Flow: 1 ml/min; column temperature 40 °C; injection volume 3 or 4 µl; UV detection at λ = 210 nm and λ = 234 nm.

1.3. Preparative Supercritical Fluid Chromatography analysis

The preparative chromatography was performed on a Supersep 150 instrument from Novasep (Pompey, France) equipped with a UV detector 40D from KNAUER GmbH (Berlin, Germany) and using the Proficy HMI/SCADA iFIX 5.1 software from GE Digital (San Ramon, CA, USA). The preparative scale column was a chiral YMC SA (250x30 mm, 5 µm particle size) from YMC CO (Kyoto, Japan).

Chiral supercritical fluid chromatography (SFC) screening and optimization was performed in a Waters Acquity UPC2 system (Waters Corp, Milford, MA, USA). The system was equipped with a photodiode array detector, a binary solvent manager and a convergence manager for mixing the supercritical CO2 with the modifier as well as a sample manager and two Acquity 30S column ovens that allowed for screening of 14 different columns in a series. The columns used in the screen were Chiralpak IB-N, Chiralpak IC, Chiralpak ID, Chiralpak IG and Chiralpak IH from Daicel (Osaka, Japan), Lux Amylose-1, Lux Cellulose-2, Lux Cellulose-3 and Lux Cellulose-4 from Phenomenex (Torrance, CA, USA), YMC SA, YMC SJ and YMC SZ from YMC CO (Kyoto, Japan), Kromasil Cellucoat from Nouryon (Bohus, Sweden) and (S,S)-Whelk-O1 from Regis Technologies Inc (Morton Grove, IL, USA). All columns measured at 150x4.6 mm with a particle size of 3 µm. The analytical system was controlled by Empower 3 software.

The analysis method used for resolution of the 4 isomers was 15% MeOH/DEA 100/20mM in CO_2 on a chiral YMC SA column.

1.4. Vibrational circular dichroism (VCD) analysis

Vibrational circular dichroism (VCD) analysis was performed in a Biotools ChiralIR2X instrument at a resolution of 4 cm^{-1} and PEM setting of 1400 cm^{-1}. The samples were dissolved in 130 µl $CDCl_3$ to give approximate concentrations of 0.025 M (P1), 0.010 M (P2), 0.074 M (P3) and 0.051 M (P4). The solutions were separately transferred to a 0.0995 mm BaF2 cell. A blank spectrum of the solvent was also acquired. Infra-red spectra were acquired on the samples concomitantly. P1, P2, and P4 are defined in Supplementary Information Section 7.2.

2. Bicyclic ketone synthesis

***N*-(But-3-en-1-yl)-4-methylbenzenesulfonamide**

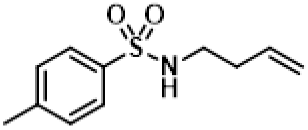

To a solution of tosyl chloride (0.953 g, 5.0 mmol, 1.0 eq.) and 3-buten-1-amine (0.46 mL, 5.0 mmol, 1.0 eq.) in Et_2O (40 mL) at 0 °C was added NEt_3 (0.77 mL, 5.5 mmol, 1.1 eq.). The reaction was stirred at rt for 4 h. The reaction mixture was filtered, washed with $NaHCO_3$ (30 mL), dried ($MgSO_4$) and concentrated to yield the title product as a colourless oil (0.86 g, 3.8 mmol, 76%). ^{1}H NMR (400 MHz, $CDCl_3$) δ 7.77 (d, *J* = 8.2 Hz, 2H), 7.34 (d, *J* = 8.2 Hz, 2H), 5.65 (ddt, *J* = 17.1, 10.3, 6.8 Hz, 1H), 5.18 – 4.98 (m, 2H), 4.38 (br s, 1H), 3.05 (q, *J* = 6.5 Hz, 2H), 2.46 (s, 3H), 2.32 – 2.17 (m, 2H); ^{13}C NMR (101

MHz, $CDCl_3$) δ 143.5, 137.0, 134.2, 129.7, 127.1, 118.3, 42.0, 33.6, 21.5. Data is consistent with reported values.

_N_-(But-3-en-1-yl)-4-methyl-_N_-(2-oxo-2-(pyrrolidin-1-yl)ethyl)benzenesulfonamide

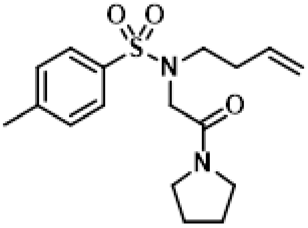

To a suspension of *N*-(but-3-en-1-yl)-4-methylbenzenesulfonamide (645 mg, 1.92 mmol, 1.0 eq.) and K_2CO_3 (637 mg, 4.61 mmol, 2.4 eq.) in MeCN (6 mL) was added 2-chloro-1-(pyrrolidin-1-yl)ethan-1-one (283 mg, 1.92 mmol, 1.0 eq.) in one portion. The reaction was stirred at rt for 18 h, then the solid was removed by filtration and the filtrate was concentrated. The residue was dissolved in EtOAc (20 mL) and washed with H_2O (20 mL). The aqueous was extracted with EtOAc (3 x 10 mL) and the combined organic extracts were dried ($MgSO_4$) and concentrated. Purification by silica gel column chromatography (Pet. Ether/EtOAc 8:2 to 7:3) yielded the title product as a colourless oil (242 mg, 0.72 mmol, 37%). ^1H NMR (400 MHz, $CDCl_3$) δ 7.76 (d, *J* = 8.2 Hz, 2H), 7.31 (d, *J* = 8.2 Hz, 2H), 5.73 (ddt, *J* = 17.1, 10.3, 6.8 Hz, 1H), 5.11 – 4.99 (m, 2H), 4.03 (s, 2H), 3.55 (t, *J* = 6.8 Hz, 2H), 3.44 (t, *J* = 7.0 Hz, 2H), 3.33 (dd, *J* = 8.5, 6.6 Hz, 2H), 2.44 (s, 3H), 2.33 (q, *J* = 7.3 Hz, 2H), 2.00 (p, *J* = 6.9 Hz, 2H), 1.87 (p, *J* = 6.8 Hz, 2H); ^{13}C NMR (101 MHz, $CDCl_3$) δ 165.9, 143.4, 136.2, 134.9, 129.5, 127.6, 117.0, 50.0, 48.0, 46.2, 46.2, 32.6, 26.3, 24.0, 21.6; HRMS calcd. for $C_{17}H_{25}N_2O_3S$ $[M+H]^+$ 337.1580, found 337.1576.

***rac*-(1*S*,5*S*)-2-Tosyl-2-azabicyclo[3.2.0]heptan-7-one (2)**

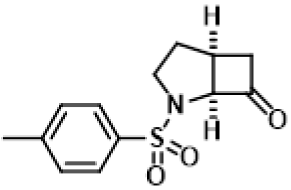

To a solution of triflic anhydride (0.18 mL, 1.08 mmol, 1.5 eq.) in DCE (9 mL) was added a solution of *N*-(but-3-en-1-yl)-4-methyl-*N*-(2-oxo-2-(pyrrolidin-1-yl)ethyl)benzenesulfonamide in DCE (9 mL) over 10 minutes. After addition was complete, a solution of 2,6-di-*tert*-butyl-4-methylpyridine (163 mg, 0.79 mmol, 1.1 eq.) in DCE (9 mL) was added dropwise over 20 minutes. The solution was heated to 90 °C for 2 h. The reaction was cooled to rt and concentrated, then the residue was dissolved in H_2O/toluene (9 mL + 9 mL). The mixture was heated to 80 °C for 2 h, then cooled to rt. The aqueous layer was extracted with CH_2Cl_2 (3 x 10 mL), the combined organics were dried ($MgSO_4$) and concentrated. Purification by silica gel column chromatography (Pet. Ether/EtOAc 1:1) yielded the title product as a yellow solid (175 mg, 0.66 mmol, 92%). ^1H NMR (400 MHz, $CDCl_3$) δ 7.79 (d, *J* = 8.3 Hz, 2H), 7.33 (d, *J* = 8.0 Hz, 2H), 5.11 (dt, *J* = 6.8, 3.4 Hz, 1H), 3.90 (ddd, *J* = 10.8, 8.2, 1.9 Hz, 1H), 3.23 – 3.13 (m, 2H), 3.13 – 3.04 (m, 1H), 2.56 (ddd, *J* = 18.1, 4.7, 3.1 Hz, 1H), 2.45 (s, 3H), 2.00 – 1.88 (m, 1H), 1.86 – 1.76 (m, 1H); ^{13}C NMR (101 MHz, $CDCl_3$) δ 205.0, 143.8, 135.9, 129.7, 127.7, 77.1, 50.3, 47.9, 30.7, 30.48, 21.6; HRMS calcd. for $C_{13}H_{16}NO_3S$ $[M+H]^+$ 266.0845, found 266.0852.

3. Diastereomers and enantiomers

Cis diastereomer

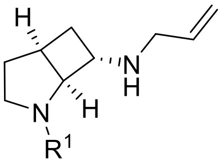

(*S,S,S*)-**3**

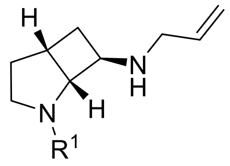

(*R,R,R*)-**3**

R^1 = Tosyl

Trans diastereomer

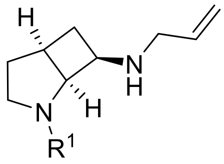

(*S,S,R*)-**3**

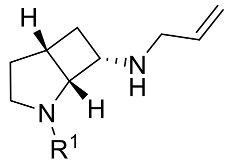

(*R,R,S*)-**3**

R^1 = Tosyl

4. Formulas and equations

Conversion and selectivity were calculated with area from supercritical fluid chromatography (SFC) chromatograms (λ = 210 or 234 nm) data using the following equations:

$$\text{Conversion (\%)} = \left(\frac{Area_{\,(all\ isomers)}}{Area_{\,(all\ stereoisomers)} + Area_{\,(byproduct)} + Area_{\,(byproduct)}}\right) * 100$$

$$d.e.(cis)\ (\%\,) = \frac{Area_{\,(cis\ diastereomer)} - Area_{\,(trans\ diastereomer)}}{Area_{\,(cis\ diastereomer)} + Area_{\,(trans\ diastereomer)}}) * 100$$

$$e.e.(S,S,S)\ (\%) = \frac{Area_{\,(S,S,S\ enantiomer)} - Area_{\,(R,R,R\ enantiomer)}}{Area_{\,(S,S,S\ enantiomer)} + Area_{\,(RR,R\ enantiomer)}}) * 100$$

$$S,S,S\ \ yield\ \ (\%) = \left(\frac{Area_{\,(S,S,S\ enantiomer)}}{Area_{\,(all\ stereoisomers)}}\right) * 100$$

$$e.e.(S,S,R)\ (\%) = \frac{Area_{\,(S,S,R\ enantiomer)} - Area_{\,(R,R,S\ \ enantiomer)}}{Area_{\,(S,S,R\ enantiomer)} + Area_{\,(R,R,S\ \ enantiomer)}}) * 100$$

$$\text{Conversion to } (S,S,S) - \mathbf{3}\ (\%) = \left(\frac{Area_{\,(S,S,S\ enantiomer)}}{Area_{\,(all\ stereoisomers)} + Area_{\,(byproduct)} + Area_{\,(substrate)}}\right) * 100$$

5. Biocatalyst gene and protein sequence

IR-09 protein sequence

MSSVSIFGLGAMGTALASRFLEEKYKVAVWNRSPEKASPLLEKGATLSHTALDGINASDLIVICLLDNAAVQATLNS
ALEHLRGKTIINLTNGTPDQARKLSDLIVSHGAQYVHGGIMATPSMIGSPHALVLYSGSPDAFKTAEADLSVLAKCIF
LGEDAGSASLHDLALLSGMYGLFSGFLHATALVRSSTPAVKFVDLLVPWLGAMTEYTKGMAKQIDEGNYASEGSN
LGMQLVAIQNIIDASAAQQVSADFIRPMKEFMEKAVVAGHGGDDISSLIDFVKST

IR-09 gene sequence

ATGTCGAGCGTAAGTATCTTCGGCTTGGGTGCTATGGGAACGGCCTTGGCTTCGCGCTTTCTGGAGGAGAAG
TACAAAGTTGCCGTGTGGAACCGTAGTCCGGAAAAGGCGTCGCCGTTACTGGAGAAAGGTGCCACGTTAAGC
CATACTGCCTTGGACGGGATCAATGCCTCGGATTTAATTGTGATTTGTCTTTTAGATAACGCCGCAGTTCAAGC
GACCCTGAACAGCGCACTGGAACACTTACGTGGTAAAACTATCATCAATCTGACGAATGGCACTCCTGACCAA
GCACGCAAACTTAGCGACTTGATTGTCTCACACGGAGCCCAATACGTGCACGGAGGAATTATGGCCACGCCTT
CTATGATTGGTTCACCACATGCATTAGTTTTATATAGCGGTTCTCCAGATGCGTTCAAAACGGCGGAAGCCGAC
CTTTCAGTGTTGGCAAAATGCATTTTCCTGGGTGAGGATGCTGGCTCCGCTTCGCTGCATGATCTTGCGCTTCT
TAGCGGAATGTATGGCTTATTCTCTGGATTCCTGCATGCTACAGCGTTAGTGCGCTCGTCAACCCCCGCGGTCA
AATTTGTGGATCTTCTGGTACCTTGGCTTGGAGCAATGACAGAGTACACAAAGGGAATGGCTAAGCAAATTG
ACGAAGGTAATTACGCCTCTGAAGGTTCCAATTTGGGTATGCAATTGGTCGCTATCCAGAACATCATCGATGC
AAGTGCAGCGCAGCAAGTAAGTGCCGACTTTATCCGTCCGATGAAAGAGTTTATGGAAAAAGCTGTTGTTGC
GGGCCACGGCGGGGACGATATTTCGAGCCTTATTGACTTCGTGAAAAGCACCTGA

IR-09 – *Aspergillus lentulus* (100% percentage identity). Annotated as “uncharacterized protein” on NCBI.

IR-20 protein sequence

MAQNSVEKAPVTLLGLGAMGTALARTWLAGGHPLTVWNRTPARAAALSPEGARVADSAAAAVAANTLVVVCLL
DDASVEEVLAGTDLADKDLVNLTTSTPAQARARAEWARERGARYLDGGIMAVPPMIGVPEIGGYVFYSGSRELFE
RHQQTLGVPAGTTYVGQDAGFAALHDVALLSAMYGMFAGAAHAFALIRKEDIDPASLAPLLADWLVAMAPTVH
QTADQLRSGDYTKGVVSSLAMQVAGTPTFLDTAAQQGVSPELLSPYFTLMRRRLAEGSAEEDLTGVIDLLVRK

IR-20 gene sequence

ATGGCCCAAAATAGCGTGGAGAAAGCTCCCGTAACATTGTTGGGACTTGGGGCGATGGGGACAGCGTTAGC
ACGTACATGGCTTGCAGGCGGCCATCCATTGACAGTCTGGAATCGCACACCTGCACGTGCCGCGGCTTTGTCA
CCAGAGGGCGCCCGCGTAGCTGACAGCGCGGCTGCGGCAGTAGCTGCTAATACATTAGTGGTCGTATGTCTT
CTTGACGACGCGAGTGTAGAAGAGGTGCTTGCGGGGACCGACCTTGCAGACAAGGACCTTGTGAACCTGACG
ACAAGTACTCCGGCACAAGCACGTGCTCGCGCAGAATGGGCTCGCGAACGTGGGGCTCGTTACCTTGACGGC
GGGATCATGGCTGTTCCCCCCATGATCGGTGTGCCGGAAATTGGCGGATATGTCTTTTACTCGGGATCGCGTG
AGTTATTTGAACGCCATCAACAAACATTGGGCGTACCTGCTGGTACAACCTACGTCGGCCAAGATGCGGGCTT
CGCCGCACTGCACGACGTAGCCTTATTATCGGCGATGTACGGTATGTTCGCCGGCGCAGCGCATGCGTTTGCC
CTGATCCGCAAAGAAGATATTGATCCGGCATCTCTTGCCCCGCTTTTGGCTGACTGGTTGGTTGCCATGGCACC
GACGGTTCATCAGACCGCCGATCAGCTTCGTTCGGGAGATTATACAAAAGGGGTTGTGAGTTCTTTAGCGATG
CAGGTGGCAGGCACTCCGACATTTTTGGACACAGCGGCGCAACAGGGCGTCAGTCCTGAGTTGCTTAGTCCTT
ATTTCACTCTGATGCGCCGCCGTCTGGCTGAAGGGTCTGCCGAAGAGGACCTGACCGGTGTGATCGACCTTCT
GGTACGCAAATAG

IR-20 – *Couchioplanes caeruleus* (100% percentage identity). Annotated as "NAD(P)-binding domain-containing protein" on NCBI.

IR-09 close homologues

IR-16 protein sequence

MSSVSIFGLGAMGKALASRFLAEKYKVAVWNRSPEKASPLLEKGATLSHTAVDGINASDLIIICLLDNAAVQATLDSA
LDRLHGKTIVNLTNGTPDQARKLSDLIVSHGAQYVHGGIMATPSMIGSPHALVLYSGSPDAFNAAEADLSVLANCV
FLGEDAGSASLHDLALLSGMYGLFSGFLHATALVKSSTPAVKFLDLLVPWLGAMTEYTKGMAKQIDEGQYASEGSN
LAMQLVAVEN IIDASAAQQVSADFIRPMKEFMEKAVAAGHGGDDISSLIDFVKST

IR-61 protein sequence

MSSVSILGLGAMGTALAARFLEKKYKVAVWNRSPEKASPLLDKGANIAQTAVDGINASDLIIICLLDNAAVQTTLAG
ALDQLQGKTIVNLTNGTPDQARKMSDLIVGHGARYVHGGIMATPSMIGSPHALVLYSGSQQAFKASEPDMLMLA
KCVFVSEDAGAASLHDLALLSGMYGLFSGFLHATALVRSSTPAVKFMDLLVPWLGAMTEYTKRMAKQIDEGNYAS
EGSNIAMQLVAIQNIIDTSAAQKVSADFIRPMKQFMEKAVAAGHSGDDISALIYFAKPS

IR-202 protein sequence

MSSVSIFGLGAMGTALASRFLEEKYKVAVWNRSPEKASSLLGKGATLSHTAVDGINASDLIIICLLDNAAVEATLAGA
LDHLHGKTIINLTNGTPDQARKLSDRFVSHGARYIHGGIMATPSMIGSPYALVLYSGSPDAFKAAEGDLSVLAKCVF
LGEDAGTASLHDLALLSGMYGLFSGFLHATALVRSSTPAVKFMDLLVPWLGAMTEYTKGMAKQIDEGKYTSEGSN
LAMQLVGIQNIIDASEAQQVSAEFIRPMKEFMQKAVAAGHGGDDISSLIDFVKST

IR-361 protein sequence

MSDPNADRPPVTVVGLGLMGQALAAAFLKGGHPTTVWNRSPEKAERLVADGAVLADTLESAVTASPLVIVCVSD
YDAVHELIRPVESALAGRVLVNLTTATSTQARETAEWAAQRNIPYLDGAIMAIPPVIGTDGAVLLYSGHKSAFEAHE
STLKAIAPAATTYLEEDHGLSSLYDMALLGIMWGILNGFLHGAALLGTAKVKAETFAPLANTMISAITEYVTAYAPQ
VDEGRYEATDAT MTVHQAAMEHLAEESEHLGIHSELPRFFKTLADRAVADGHAENSYAAMIELFRKPTA

IR-09 close homologues were identified from the phylogenetic tree of the metagenomic panel. This phylogenetic tree can be found in https://doi.org/10.1038/s41557-020-00606-w Supplementary Information Section 4.2.

6. Procedure for the production of biocatalysts

6.1. Origin and cloning

A metagenomic panel of 384 IREDs was provided by Prozomix, small samples of these enzymes were obtained and expressed using the same conditions as outlined in https://doi.org/10.1038/s41557-020-00606-w Supplementary Information Section 3.

6.2. Expression

IR-09 WT and variants D167, W204A, W204S, M233A and Q234A were expressed in house to obtain larger quantities of catalyst. BL21 (DE3) glycerol stock was streak on lysogeny broth agar (LBA) plate

supplemented with 50 µg/mL kanamycin to grow overnight at 37 °C, single colony was picked and grown overnight in 15 ml LB medium supplemented with 50 µg/mL kanamycin. Next, the preculture was used to inoculate 400 ml of terrific broth (TB) supplemented with 1.6 mL glycerol (60% v/v) and with 50 µg/mL kanamycin. Flasks were grown at 37 °C for 1.5 hrs at 200 rpm and then induced with 0.1 mM IPTG. For expression, flaks were incubated at 23 °C for 24 hrs at 200 rpm. The cultures were transferred into 500 mL centrifuge pots and centrifuged at 8500 g, 4°C for 25 min to recover the cells. Pellets were stored at -20°C .

6.3. Cell-free enzyme (CFE) preparation

Pellets were then placed into sterile 50 mL Falcons and resuspended in 0.1 M sodium phosphate buffer (pH 7.2) to remove remaining TB. Cells were centrifuged at 8500 g, 4°C for 25 min and were resuspended again in 0.1 M sodium phosphate buffer (pH 7.2) for sonication. Cells were sonicated for 30 seconds on and 45 seconds off at 20-25 microns for 7 cycles. Next, samples were centrifuged at 18000 rpm for 40 minutes at 4°C. Supernatant was recovered and frozen for lyophilisation, which was performed at 0.05 mbar for 48 hours. Lyophilised cell-free extract was stored at -20°C. 10 µL of supernatant was recovered prior to lyophilisation to be analysed by SDS-PAGE to confirm expression.

6.4. Biocatalyst purification

After protein expression, IR-09WT pellets were resuspended in 30 mL of 100 mM KPi pH 7.8 buffer containing 300 mM NaCl and 20 mM imidazole (buffer A), another 100 mM KPi pH 7.8 buffer containing 300 mM NaCl and 1 M imidazole (buffer B) was prepared for later use. Cells were sonicated for 30 seconds on and 99 seconds off at 20 microns for 7 cycles. Next, the suspension was centrifuged at 18000 rpm for 20 minutes at 4°C and 10 µL of supernatant were recovered prior to purification to be analysed by SDS-PAGE to confirm expression. Supernatant was filtered using 0.45 µm filters and loaded onto a 5 mL His-Trap FF column (GE Healthcare) charged with 0.1 M nickel sulphate and equilibrated with A. The protein was then eluted using an ÄKTA purifier (GE Healthcare) through a two-step method which consisted of a 10 minute wash with buffer A and a gradient program starting from 0% buffer B up to 100% buffer B, both steps were performed at 5 mL/min and collected in 5 mL fractions. The fractions were analysed for protein content by SDS page and fractions containing protein were concentrated and buffer exchanged into 100mM TRIS buffer, pH 8.0, by spin column (Vivaspin 20, 10 kDa cutoff, GE Healthcare) and gravity column. The protein was then snap-frozen using liquid N2 before storage at -80°C.

7. General procedure for biotransformations

The colorimetric high throughput screening was performed with protocol outlined in https://doi.org/10.1038/s41557-020-00606-w Supplementary Information Section 4.2.

Potential hits were corroborated by performing the reaction on the reductive direction, analytical scale reductive amination biotransformations were carried out at 500 µl scale adjusted to pH 8.0 containing 4 mg/ml lyophilised powder of supernatant of lysate IRED, 0.5 mg/ml GDH (Codexis CDX-901), 0.5mM $NADP^+$ (Prozomix), 40 mM D-glucose, 5% DMSO, 10 mM rac-**2** and 10 equivalences of allylamine (1 M allylamine stock pH 8.0 adjusted), with the reaction volume made up to 500 µL in 100 mM Tris Buffer. The reaction mixture was incubated at 37°C with shaking at 900 rpm (Eppendorf

ThermoMixer) for 20 hours (overnight). Following 20 hours the reaction was quenched by addition of 20 µL of 10 M NaOH. Then, 1 volume of methyl tert-butyl ether (MTBE) was added for extraction, oraginc layer was recovers and extraction was repeated. Both organic layers (MTBE) were mixed and dried with magnesium sulphate ($MgSO_4$). The mixture was centrifuged for 5 minutes at 13300 rpm and supernatant was recovered to be analysed by supercritical fluid chromatography (SFC).

7.1. Amine equivalences optimization

IR-09WT and variants W204S and W204R conversion were studied with lower amine equivalences. One of the key differences between RedAms and IREDS is that RedAms also catalyse imine formation in the active site, therefore RedAms can perform reductive amination in equimolar conditions while IREDS require larger amine concentrations. Biotransformations were performed at 500 µl scale adjusted to pH 8.0 containing 4 mg/ml lyophilised powder of supernatant of lysate IRED, 0.5 mg/ml GDH (Codexis CDX-901), 0.5mM $NADP^+$ (Prozomix), 40 mM D-glucose, 5% DMSO, 10 mM rac-2 and 1, 2 5 or 10 equivalents of allylamine (1 M allylamine stock pH 8.0 adjusted), with the reaction volume made up to 500 µL in 100 mM Tris Buffer. The reaction mixture was incubated at 37°C with shaking at 900 rpm (Eppendorf ThermoMixer) for 20 hours (overnight). Then, 1 volume of methyl tert-butyl ether (MTBE) was added for extraction, organic layer was recovers and extraction was repeated. Both organic layers (MTBE) were mixed and dried with magnesium sulphate ($MgSO_4$). The mixture was centrifuged for 5 minutes at 13300 rpm and supernatant was recovered to be analysed by supercritical fluid chromatography (SFC).

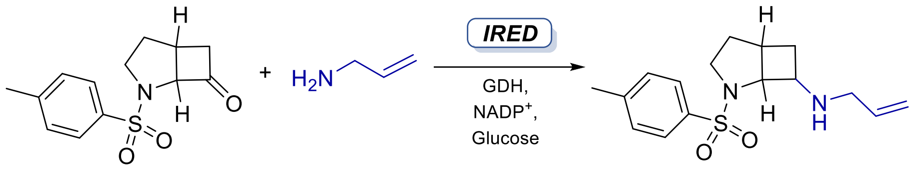

rac-**2** **1** **3**

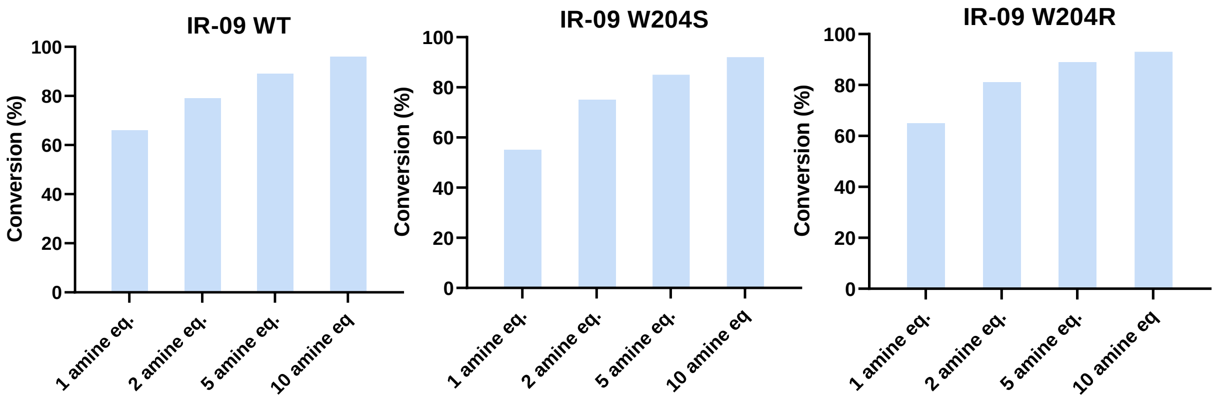

Figure S1. Reductive amination of rac-**2** with **1** with lower amine equivalences. Reaction conditions: 4 mg·ml^{-1} IRED(CFE), 40 mM Glucose, 0.5 mM $NADP^+$, 0.5 mg·ml^{-1} GDH, 100 mM Tris pH 8, 37 °C, 24 h.

IR-09WT and these two variants proved to perform reductive amination in equimolar amine concentration.

7.2. IR-09 close homologues

Table S1. Reductive amination of rac-**2** with **1** with IR-09 close homologues and IR-20. Reaction conditions: 4 mg·ml^{-1} IRED (CFE), 40 mM Glucose, 0.5 mM $NADP^+$, 0.5 mg·ml^{-1} GDH, 100 mM Tris pH 8, 37 °C, 24 h.

IRED	Conversion (%)	*d.e.* cis (%)	*e.e.* (S,S,S) (%)	S,S,S ratio (%)
IR-09	96	-85	-99	0
IR-16	89	-94	-99	0
IR-61	51	-98	-99	0
IR-202	74	-92	-99	0
IR-374	34	-99		0
IR-20	36	-99		0

7.3. IR-09 variants

Table S2. Reductive amination of rac-**2** with **1** with IR-09 variants. Same reaction conditions as Table S1.

IRED	Conversion (%)	*d.e.* cis (%)	*e.e.* (S,S,S) (%)	S,S,S ratio (%)
IR-09 WT	96	-85	-99	0
IR-09 D167Y	24	-99		0
IR-09 M233A	81	-93	-99	0
IR-09 Q234A	64	-99		0
IR-09 W204A	92	-48	64	21
IR-09 W204S	92	-39	80	28
IR-09 W204L	89	-47	85	19
IR-09 W204G	75	15	96	56
IR-09 W204R	93	-9	95	45

8. Primers sequences

Table S3. Primers to set up IR-09 site directed variants.

Name	Sequences
IR-09_D176Y_f	CCGCTAAGAAGCGCAAGATAATGCAGCGAAGCGGAGC
IR-09_D176Y_r	GCTCCGCTTCGCTGCATTATCTTGCGCTTCTTAGCGG
IR-09_W204A_f	CATTGCTCCAAGCGCAGGTACCAGAAGATCC
IR-09_W204A_r	GGATCTTCTGGTACCTGCGCTTGGAGCAATG
IR-09_W204S_f	CATTGCTCCAAGGCTAGGTACCAGAAGATCC
IR-09_W204S_r	GGATCTTCTGGTACCTAGCCTTGGAGCAATG
IR-09_M233A_f	CTGGATAGCGACCAATTGCGCACCCAAATTGGAACCTTC
IR-09_M233A_r	GAAGGTTCCAATTTGGGTGCGCAATTGGTCGCTATCCAG
IR-09_Q234A_f	CTGGATAGCGACCAACGCCATACCCAAATTGGAACCTTC
IR-09_Q234A_r	GAAGGTTCCAATTTGGGTATGGCGTTGGTCGCTATCCAG

Table S4. Primers to set up IR-09 site saturated variants.

Name	Sequences
IR-09_W204NNK_f	GTGGATCTTCTGGTACCTNNKCTTGGAGCAATGAC
IR-09_W204NNK_r	GTCATTGCTCCAAGMNNAGGTACCAGAAGATCCAC

9. Crystallization **IR-09**

Table S5. Data collection and refinement statistics.

	IR-09 – **8QHE**
Wavelength	0.9688
Resolution range	36.89 - 1.6 (1.657 - 1.6)
Space group	P 21 21 2
Unit cell	79.26 101.10 36.39 90 90 90
Total reflections	488799 (46659)
Unique reflections	39488 (3920)
Multiplicity	12.4 (11.9)
Completeness (%)	99.81 (99.16)
Mean I/sigma(I)	12.24 (1.16)
Wilson B-factor	31.34
R-merge	0.0852 (2.317)
R-meas	0.0891 (2.423)
R-pim	0.0256 (0.7023)
CC1/2	0.998 (0.536)
CC*	0.999 (0.835)
Reflections used in refinement	39419 (3890)
Reflections used for R-free	2019 (191)
R-work	0.1816 (0.2965)
R-free	0.2116 (0.3132)
CC(work)	0.970 (0.738)
CC(free)	0.958 (0.806)
Number of non-hydrogen atoms	2350
macromolecules	2134
ligands	98
solvent	159
Protein residues	284
Nucleic acid bases	
RMS(bonds)	0.02
RMS(angles)	1.69
Ramachandran favored (%)	97.52
Ramachandran allowed (%)	2.48
Ramachandran outliers (%)	0
Rotamer outliers (%)	1.75
Clashscore	5.02
Average B-factor	40.35
macromolecules	40.36
ligands	30.43
solvent	43.72
Number of TLS groups	7

Single crystals of IR-09 were grown by sitting drop vapour diffusion by mixing equal volumes (200nl) of protein and a reservoir solution comprised of 0.2 M Magnesium chloride hexahydrate 0.1 M Tris

8.5 20 % w/v PEG 8000 (JCSG D6). Drops were incubated at 4°C and crystals grew over a period of 24 hours. Crystals were cryo-protected with the addition of PEG 200 to the mother liquor prior to flash cooling in liquid nitrogen. Single crystal diffraction data were collected at Diamond Light Source and subsequently reduced using DIALS and Xia2. The structure was solved by molecular replacement using a search model derived from (6EOI) and subsequently rebuilt and refined in COOT and Phenix.refine respectively. Validation with MolProbity and PDB-REDO were integrated in the iterative rebuild and refinement process. Final resolution was determined using paired refinement in PDB-REDO. Complete data collection and refinement statistics are presented in Table S5. PDB deposition code: **8QHE**

10. Docking

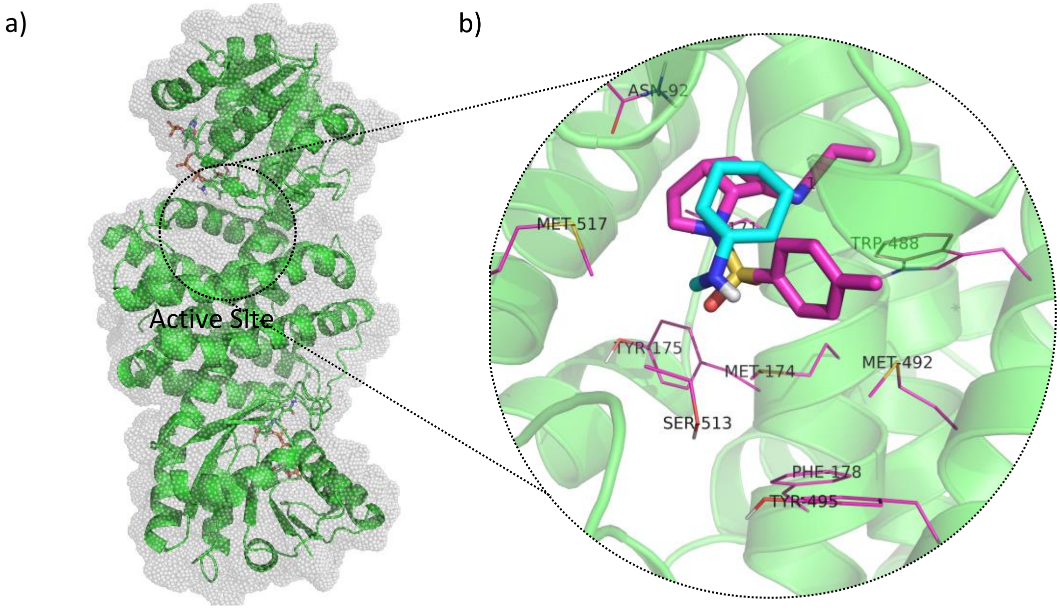

Figure S2. a) Representative structure of dimer taken from 50ns of MD simulation. B) Docked poses of imine intermediate **3** in magenta and co-crystal N-cyclopropylcyclohexanamine in blue. Shown in sticks are the residues selected for flexibility. The active site of chain A was chosen for docking.

A model was built based on the monomeric crystal structure of {protein name} with bound {co-crystal name} and NADP. A biochemical dimer was constructed using a pdb of the monomer and its identical symmetry neighbour. The protonation states of titratable residues were calculated using ProPKA3. The Amber FF14SB force field was used, and General Amber Force-Field (GAFF) with NADP and {substrate name} parameters were generated using using Antechamber with charges obtained by RESP fitting to a HF/6-31G(d,p) single-point calculation on a structure optimized at the B3LYP/6-31G (d,p)//PCM(water) level of theory, using Gaussian09 revision D41.

The system was solvated in a TIP3P water box of at least 10 Å around the protein, and counter-ions added to neutralize the system charge. Calculations were then performed using Gromacs 2016. A stepwise energy minimization protocol was utilized, with a decreasing degree of positional restraints: (i) everything except solvent and ions restrained; (ii) restraints on hydrogen atoms were removed; (iii) all restraints removed.

After energy minimization, the solvent was equilibrated for 100 ps using the constant-volume NVT ensemble with positional restraints applied to the protein, cofactor and substrates. The same progressive scheme of positional restraints as during energy minimization was then applied to

constant-pressure equilibration during successive 100 ps constant-pressure NPT ensemble simulations.

Molecular docking simulations were performed using AutoDockTools software suite (version 4.6.1) to assess the binding affinities of the ligands to the receptor. The receptor structure was obtained from a 50 ns molecular dynamics (MD) simulation, and the representative structure was chosen based on the average active site conformation. In the preparation step, non-polar hydrogens were merged, and Gasteiger charges were added. All water molecules were removed from the receptor structure.

The ligands were drawn and optimized using ChemDraw (version 20.0) and subsequently converted to PDB mat and energy minimised using AnteChamber. The docking was performed allowing flexibility for all residues within a 5 Å radius around the representative substrate pose obtained from MD. The grid box for docking was set to include the entire active site and any potentially interacting residues. The dimensions of the grid box were defined based on the extent of the active site and its surrounding flexible residues.

AutoDock Vina was run with an exhaustiveness parameter of 50 to ensure a sufficient sampling of the conformational space. The docking protocol generated 12 poses for each ligand, and these poses were ranked based on their predicted binding affinities. The docking pose chosen to inform mutagenesis work was pose 4, which has the lowest energy whilst still demonstrating interactions in the known chemistry.

11. Procedure for mutagenesis

11.1. Site directed mutagenesis (SDM)

Single point variants were prepared with QuickChange site directed mutagenesis kit (Agilent) and with corresponding primers for each variant. Primers were designed with approximately 15 bp overhangs and if possible, ending with G or C. Primers were obtained from Eurofins. Tm were calculated with SnapGene. Variants were sequenced to confirm mutagenesis.

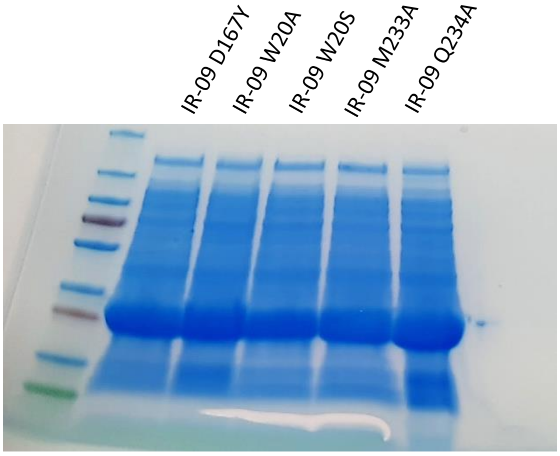

Figure S3. SDS PAGE analysis of IR-09 site directed variants (lysate).

11.2. Site saturation mutagenesis (SSM)

Site saturation variants were prepared with degenerated codon (NNK and NNM). Primers were designed with approximately 15 bp overhangs and if possible, ending with G or C. Primers were

obtained from IDT. Tm were calculated with SnapGene. Library was sequenced and it exhibited excellent base distribution for the targeted codon. After screening, best variants were sequenced to identify the side chain.

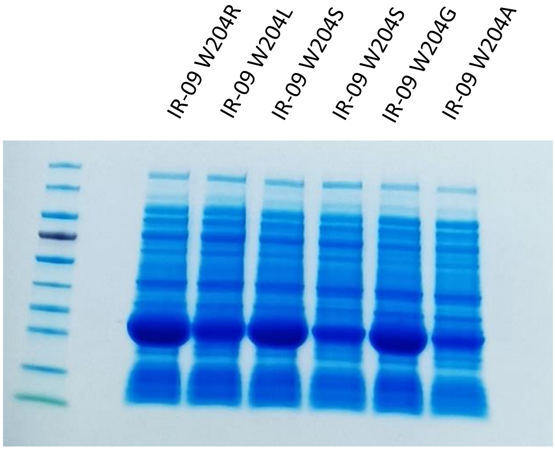

Figure S4. SDS PAGE analysis of IR-09 site saturated variants (lysate). Variants with highest *e.r.* for (S,S,S)-**3** were expressed in 2 L flasks (400 mL) and were rescreened to corroborated improved stereoselectivity.

12. Preparative-scale reaction

IR-09 WT and IR-09 W204R were used for preparative-scale reactions (50 mLs) on EasyMax. Racemic starting material was synthesised in house.

Preparative-scale reductive amination biotransformations were carried out at 50 mL scale adjusted to pH 8.0 containing 4 mg/ml lyophilised powder of supernatant of lysate IRED, 0.5 mg/ml GDH (Codexis CDX-901), 0.5mM $NADP^+$ (Prozomix), 40 mM D-glucose, 5% DMSO, 10 mM rac-**2** and 10 equivalences of allylamine (1 M allylamine stock pH 8.0 adjusted), with the reaction volume made up to 50 mL in 100 mM Tris Buffer. The reaction mixture was incubated at 37°C with shaking at 900 rpm (Eppendorf ThermoMixer) for 20 hours (overnight). Following 20 hours the reaction was quenched by addition of 2 mL of 10 M NaOH. Then, 1 volume of methyl tert-butyl ether (MTBE) was added for extraction, organic layer was recovers and extraction was repeated. Both organic layers (MTBE) were mixed and dried with magnesium sulphate ($MgSO_4$). The mixture was filtered and concentrated under *vacuo*. The crude was shipped to AstraZeneca facilities, where it was dissolved in methanol and separation of the four isomers was performed on preparative SFC (10% MeOH/DEA 100/20mM in CO_2, at 125 bar back pressure and 40 °C), using a YMC SA chiral column (250x30mm, 5 µm).

IR-09 WT and IR-09 W204R exhibited 95% and 91% conversions, respectively. Stereoselectivity was the same as in analytical scale.

13. Procedure for deallylation

(1*S*,5*S*,7*S*)-2-Tosyl-2-azabicyclo[3.2.0]heptan-7-amine

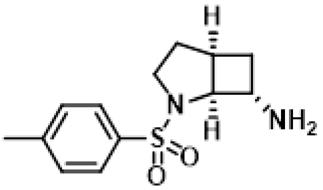

A solution of Pd(dba)$_2$ (1.3 mg, 0.002 mmol, 5 mol%) and DPPB (1.0 mg, 0.002 mmol, 5 mol%) in THF (0.25 mL) was stirred at rt for 15 minutes. The catalyst solution and thiosalicyclic acid (7.7 mg, 0.05 mmol, 1.1 eq.) were added to a solution of (1*S*,5*S*)-*N*-allyl-2-tosyl-2-azabicyclo[3.2.0]heptan-7-amine **3** (14 mg, 0.046 mmol, 1.0 eq.) in THF (0.25 mL). The reaction was heated to 60 °C and stirred for 2 h, then cooled to rt and 1M HCl (5 mL) was added. The aqueous was washed with EtOAc (3 x 5 mL) and the washings were discarded, then 2M NaOH was added. The aqueous was extracted with EtOAc (3 x 5 mL), the combined organics were dried (MgSO$_4$) and concentrated to yield the title product as a colourless oil (9.0 mg, 0.034 mmol, 73%). ^{1}H NMR (400 MHz, CDCl$_3$) δ 7.72 (d, *J* = 8.4 Hz, 2H), 7.31 (d, *J* = 8.1 Hz, 2H), 4.24 (td, *J* = 5.8, 2.9 Hz, 1H), 3.76 – 3.60 (m, 3H), 2.87 (br s, 2H), 2.72 (p, *J* = 7.3 Hz, 1H), 2.54 – 2.46 (m, 1H), 2.44 (s, 3H), 1.65 – 1.43 (m, 2H), 1.40 – 1.29 (m, 1H); ^{13}C NMR (126 MHz, CDCl$_3$) δ 143.6, 135.7, 129.8, 127.2, 64.8, 50.0, 46.6, 34.3, 32.7, 29.6, 21.5 ppm; HRMS calcd. for $C_{13}H_{19}N_2O_2S$ $[M+H]^+$ 267.1162, found 267.1161.

14. Chromatograms and MS spectra

14.1. Analytical SFC chromatograms

14.1.1. Wild Type enzymes

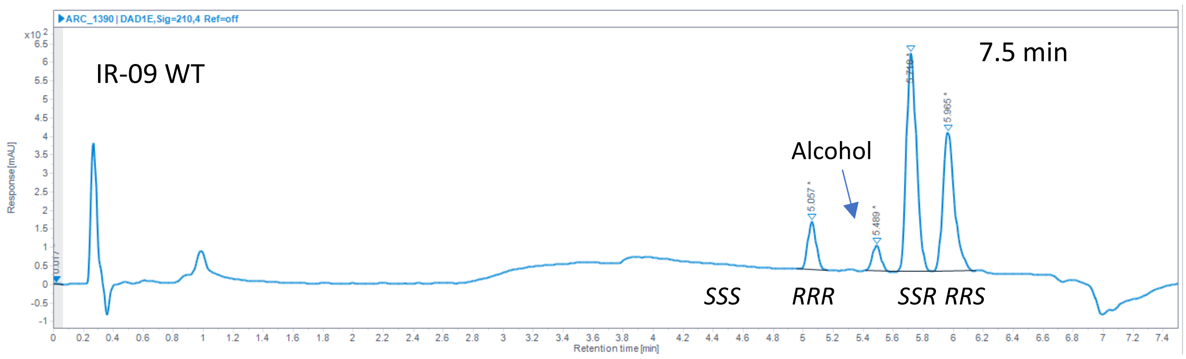

Figure S5. IR-09 WT SFC chromatogram with a 7.5 method.

14.1.2. Pure IR-09

All biotransformations were performed with cell free enzyme (CFE) and GDH as NADPH$^+$ recycling system, all of them produced small quantities of the corresponding alcohol as by-product. IRED are usually highly chemoselective and it has been reported in literature that alcohol formation is usually because of KREDs in the CFE and GDH. Therefore IR-09 WT was purified and biotransformation were

performed with pure IR-09 with GDH and without (w/o) GDH as NADPH+ recycling system. Results confirm that KREDs present in CFE and GDH were the enzymes to produce the ketone reduction to the alcohol, pure IR-09 WT without GDH didn't yield any alcohol formation.

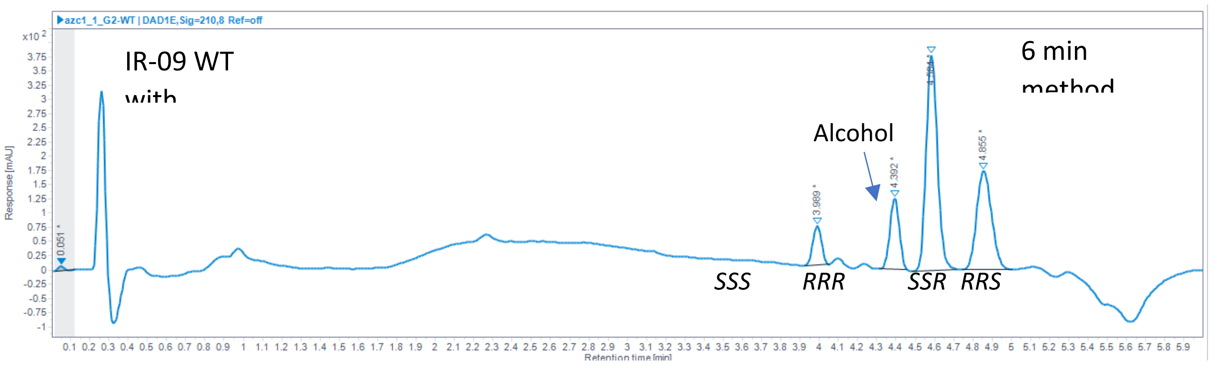

Figure S6. IR-09 WT with GDH as NADPH+ recycling system SFC chromatogram with a 6 min method.

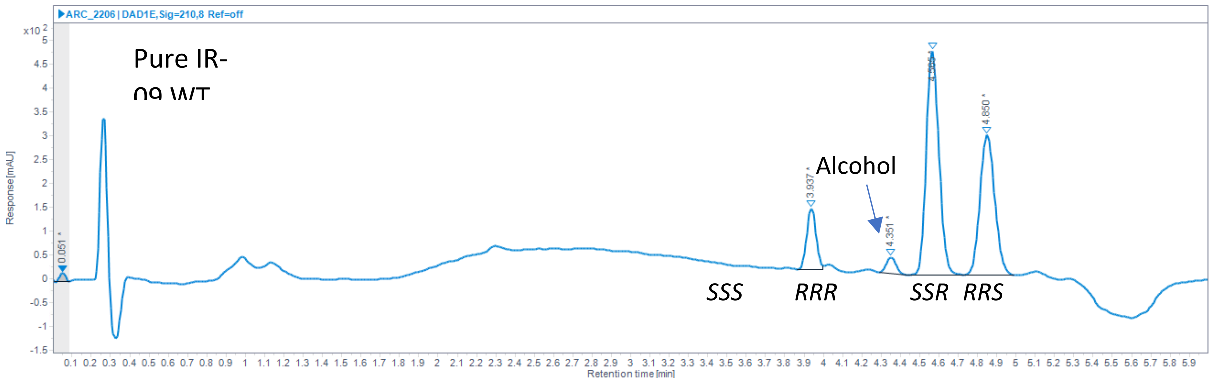

Figure S7. Pure IR-09 WT with GDH as NADPH+ recycling system SFC chromatogram with a 6 min method. Alcohol formation was considerably lower.

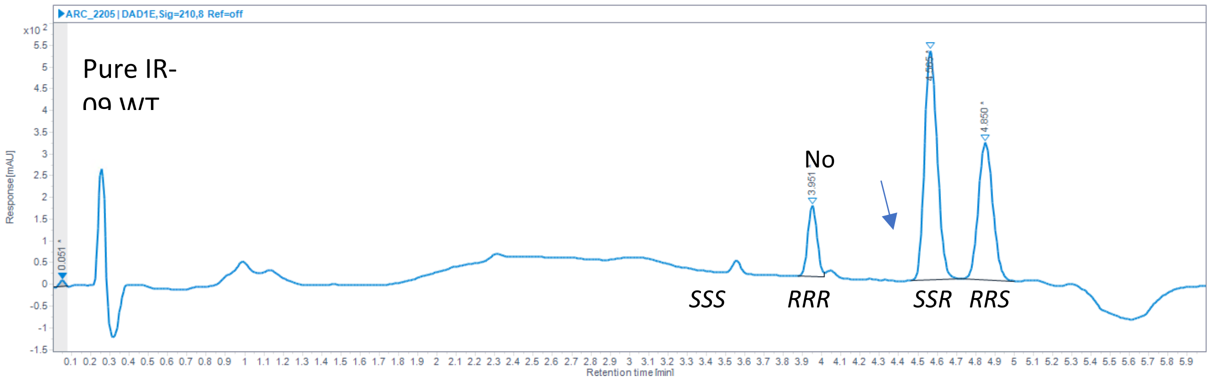

Figure S8. Pure IR-09 WT without GDH as NADPH+ recycling system SFC chromatogram with a 6 min method. There was no alcohol formation.

14.1.3. Site directed mutagenesis (SDM) variants

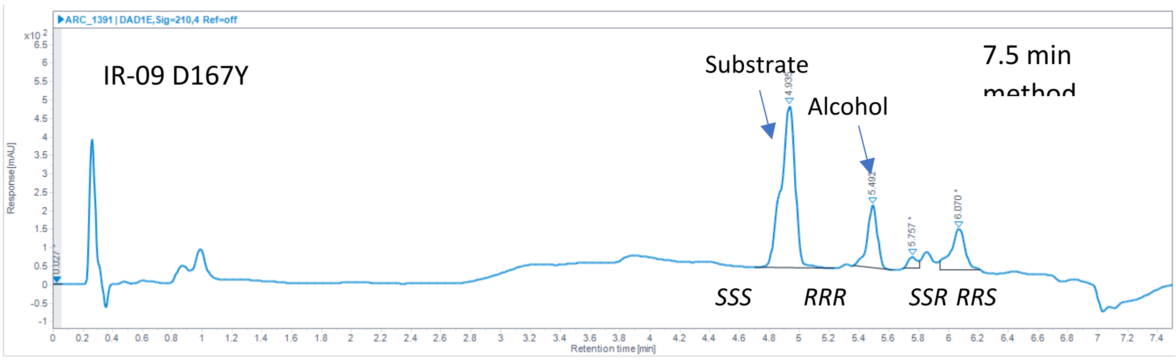

Figure S9. IR-09 D167Y SFC chromatogram with a 7.5 min method. D169Y exhibits a big loss of activity.

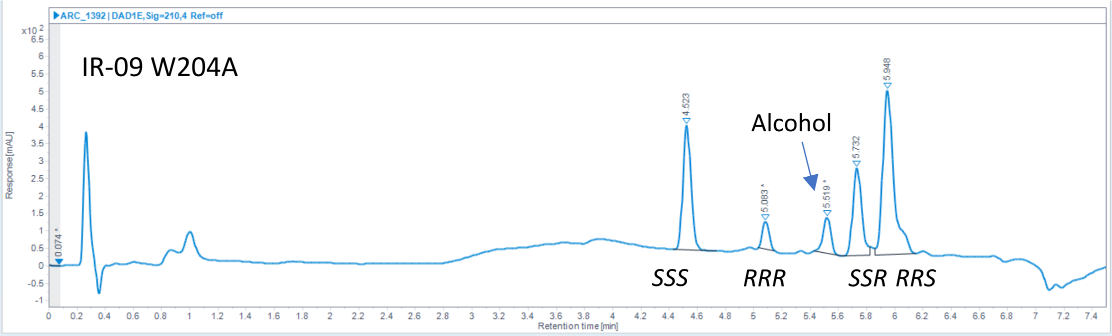

Figure S10. IR-09 W204A SFC chromatogram with a 7.5 min method. This variant could produce the (S,S,S)-enantiomer.

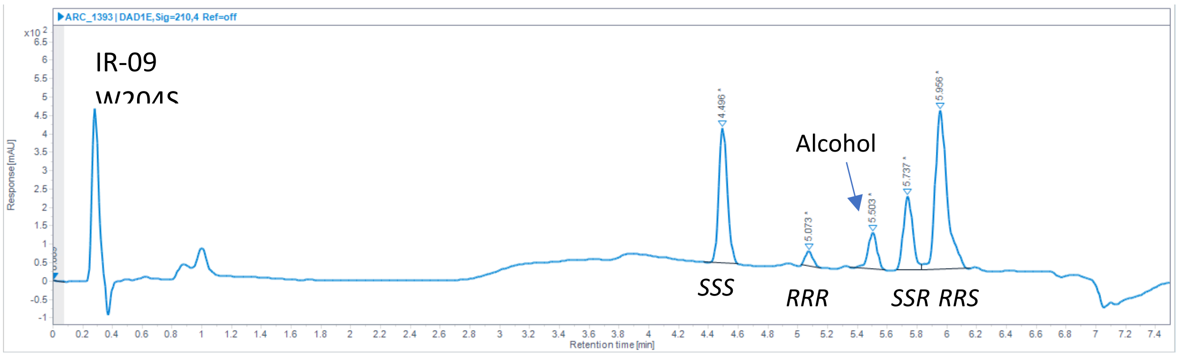

Figure S11. IR-09 W204S SFC chromatogram with a 7.5 min method. This variant could produce the (S,S,S)-enantiomer.

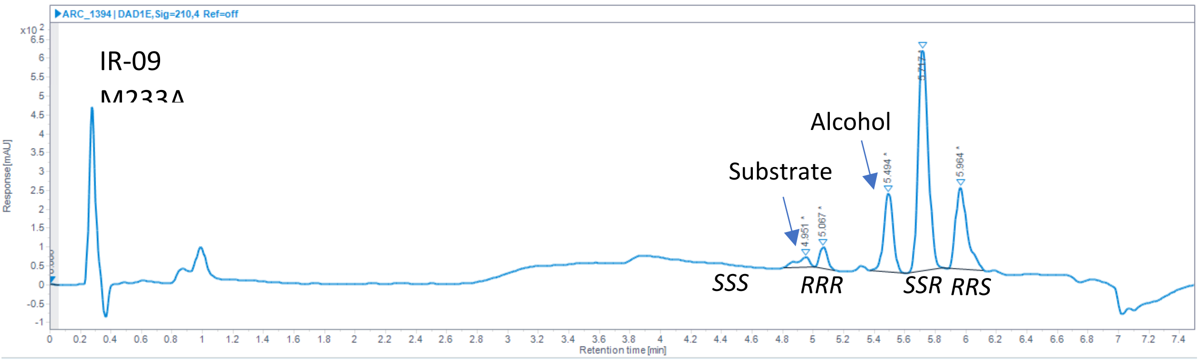

Figure S12. IR-09 M233A SFC chromatogram with a 7.5 min method.

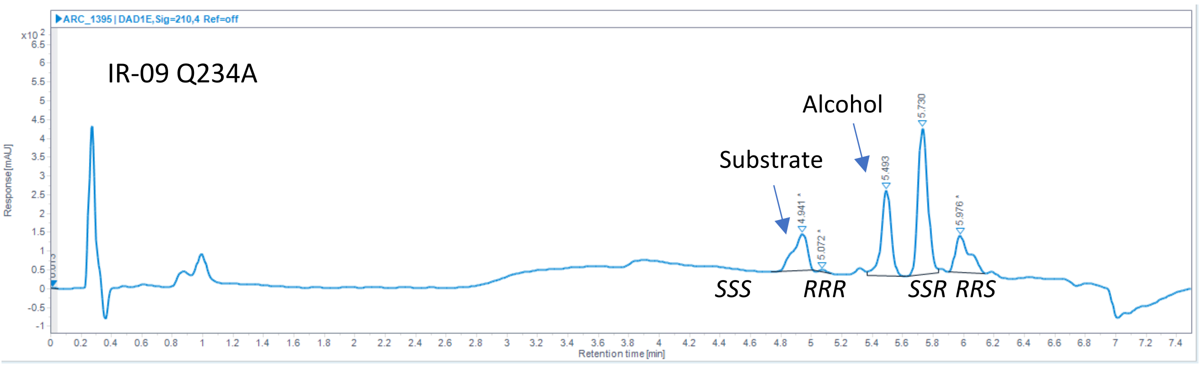

Figure S13. IR-09 Q234A SFC chromatogram with a 7.5 min method.

14.1.4. Site saturated mutagenesis (SSM) variants

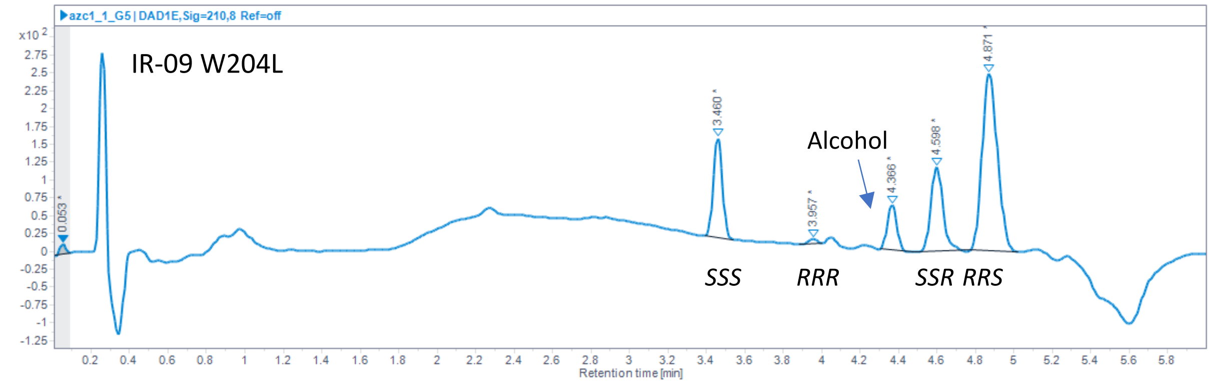

Figure S14. IR-09 W204L SFC chromatogram with a 6 min method.

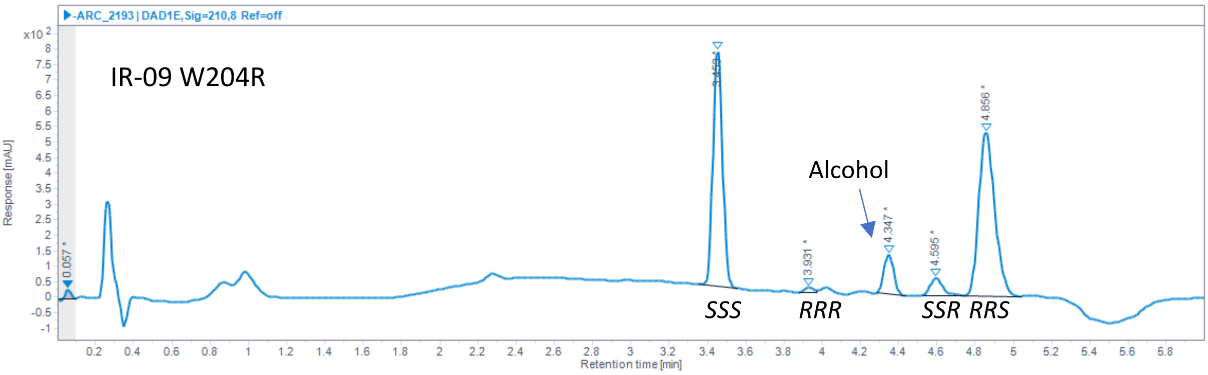

Figure S15. IR-09 W204R SFC chromatogram with a 6 min method.

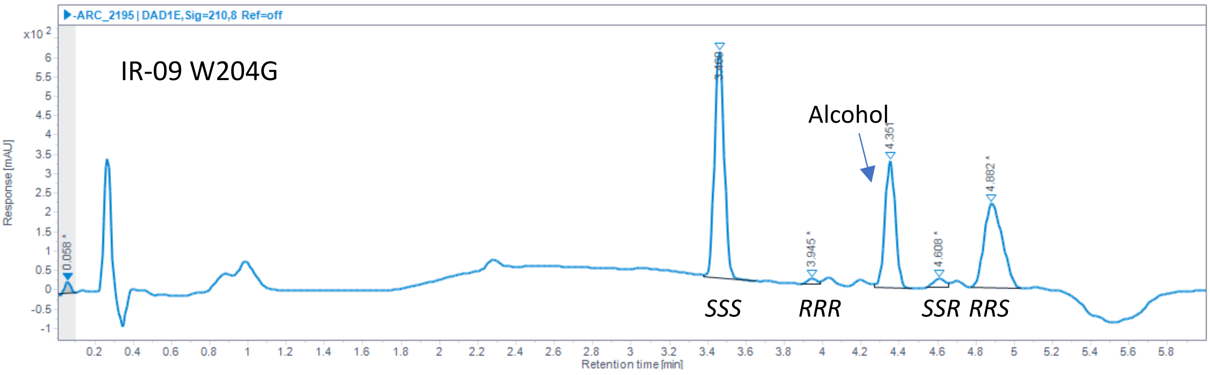

Figure S16. IR-09 W204G SFC chromatogram with a 6 min method.

14.2. Preparative SFC chromatogram and MS spectrums

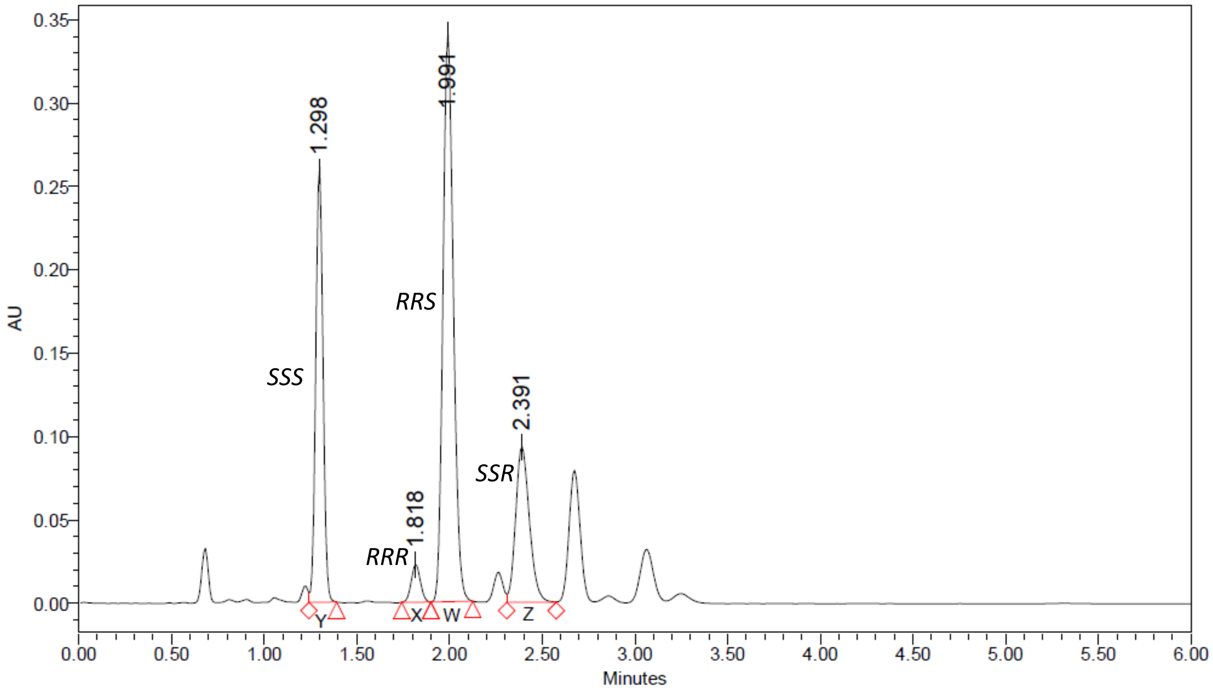

Figure S17. IR-09 W204S preparative SFC chromatogram with a 6 min method.

Note that preparative SFC was done with a different column and solvents as analytical SFC. This led to changes in retention times. Furthermore, these new conditions switch elution times for trans diastereomer, *RRS* was eluting earlier than *SSR*. For the analytical SFC, the *SSR* enantiomer eluted earlier than *RRS* enantiomer. This process was performed by different teams and different instrument, this led to different names for each peak. See below a table with a summary of different nomenclatures.

Table S6. Summary of different nomenclatures

Absolute configuration	Letter nomenclature	"P" nomenclature (VCD)
S,S,S	Y	P1
R,R,R	X	P2
S,S,R	Z	P4
R,R,S	W	P3

Purified enantiomers with MS spectrum

P1 (*S,S,S*)

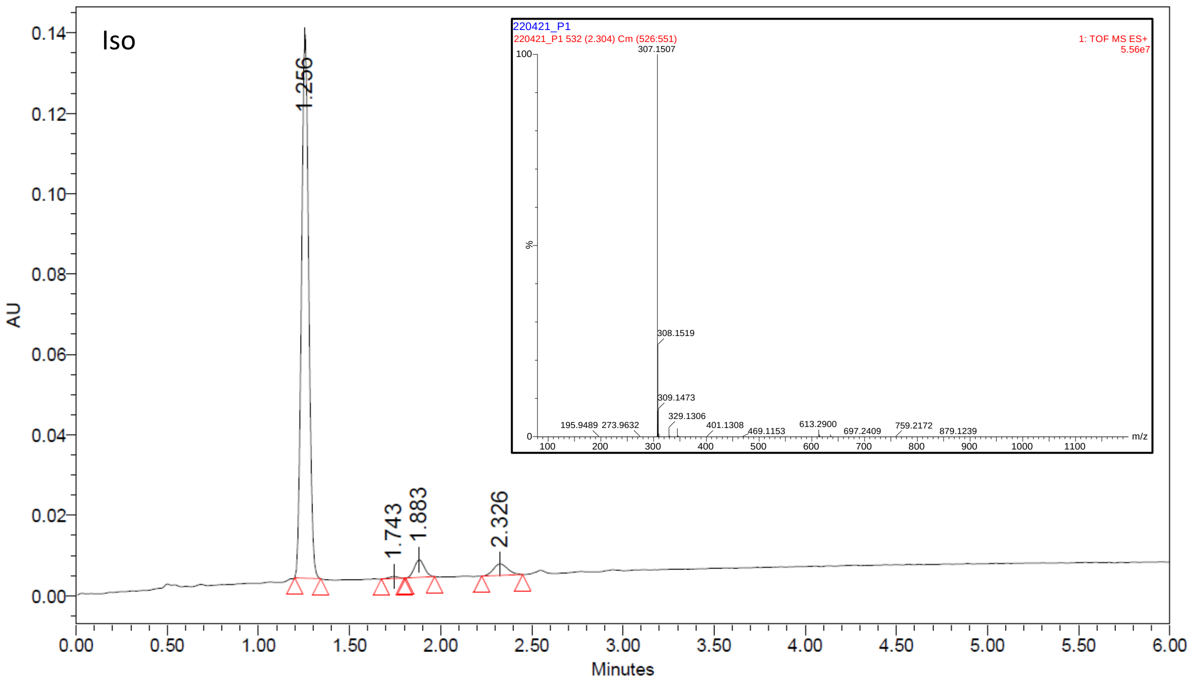

Figure S18. Purified P1 (*S,S,S*) with MS spectrum (product+H)$^{+}$. Product correspondent to IR-09 M204S recovered by preparative SFC.

P2 (*R,R,R*)

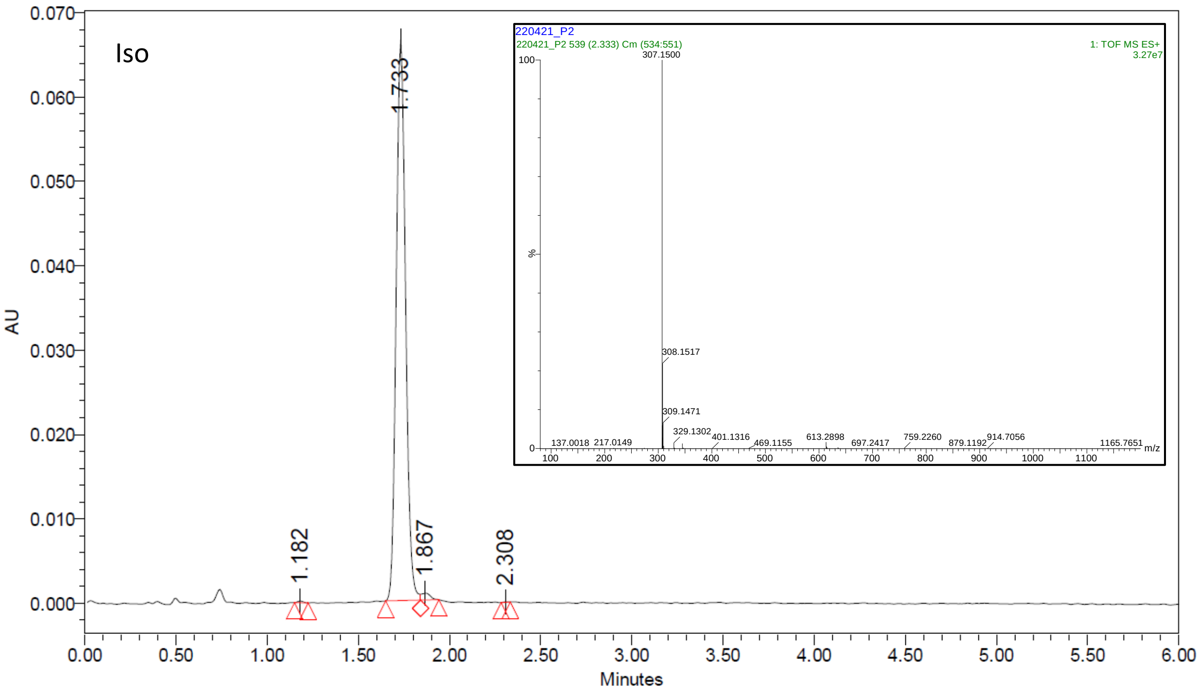

Figure S19. Purified P2 (*R,R,R*) with MS spectrum (M+H)$^{+}$. Product correspondent to IR-09 W204S recovered by preparative SFC.

P3 (*R,R,S*)

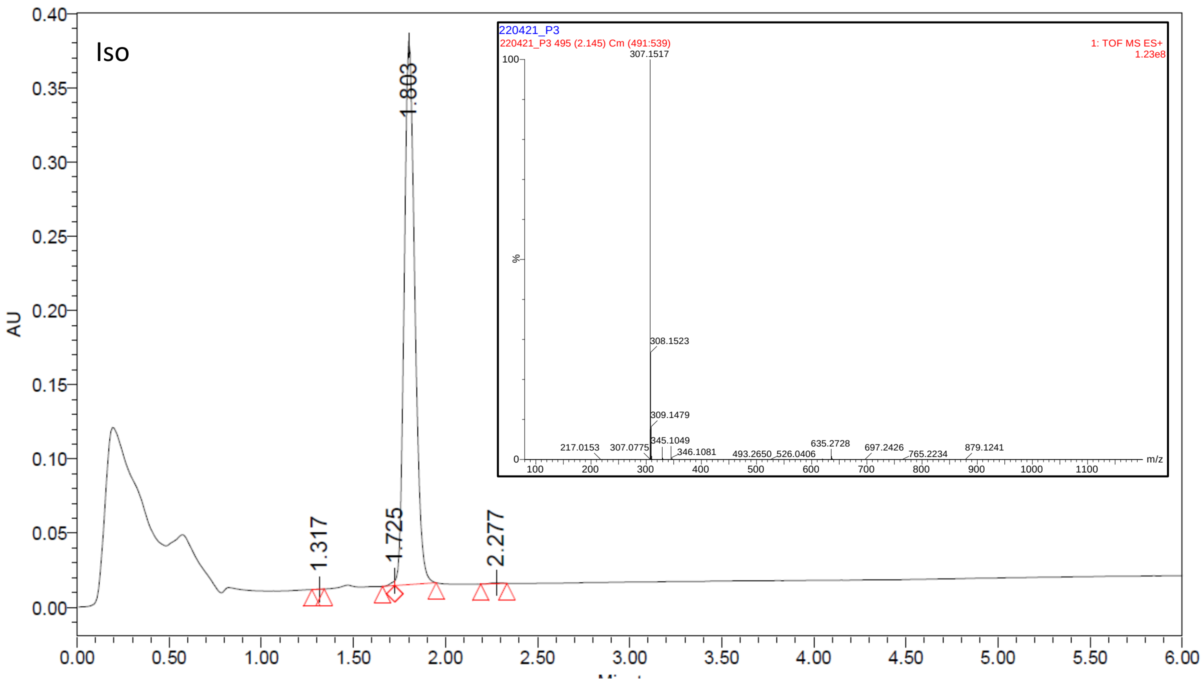

Figure S20. Purified P3 (*R,R,S*) with MS spectrum (M+H)$^+$. Product correspondent to IR-09 W204S recovered by preparative SFC.

P4 (*S,S,R*)

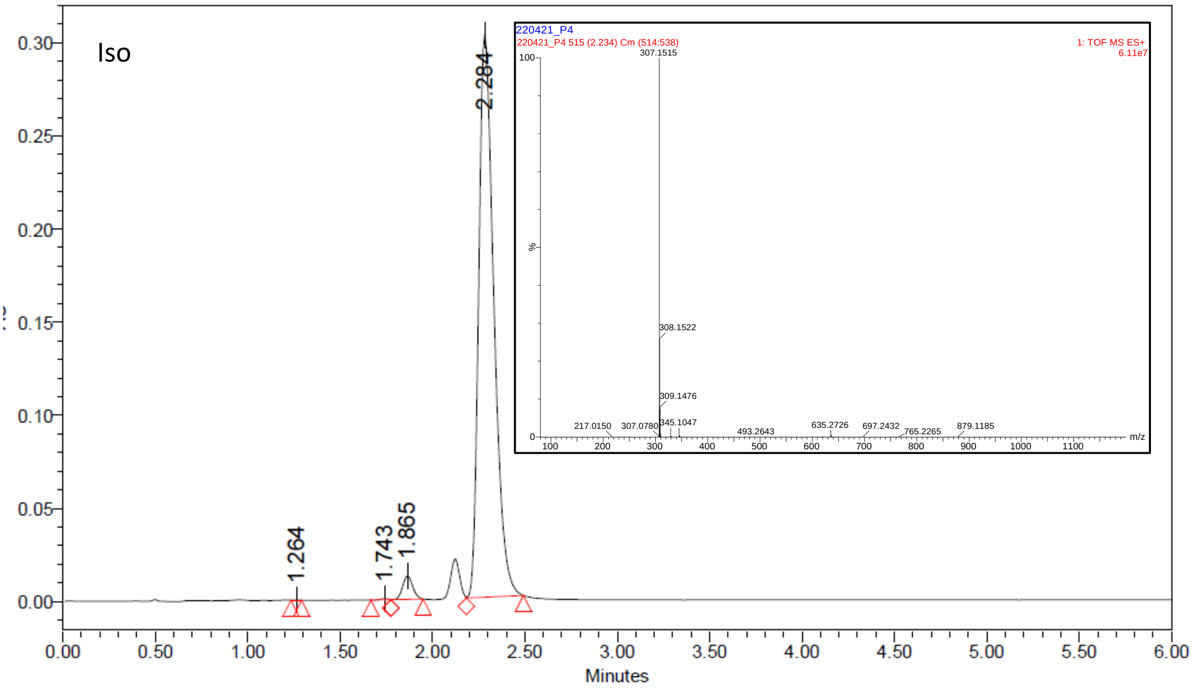

Figure S21. Purified P4 (*S,S,R*) with MS spectrum (M+H)$^+$. Product correspondent to IR-09 W204S recovered by preparative SFC.

15. Vibrational circular dichroism (VCD) results

15.1. Experimental infra-red and VCD spectra

The experimental infra-red and VCD spectra are shown in Figures S1 and S2. Note that the IR spectra of P1-P4 are very similar to each other, though there are low level impurity peaks visible in the IR spectrum of P2.

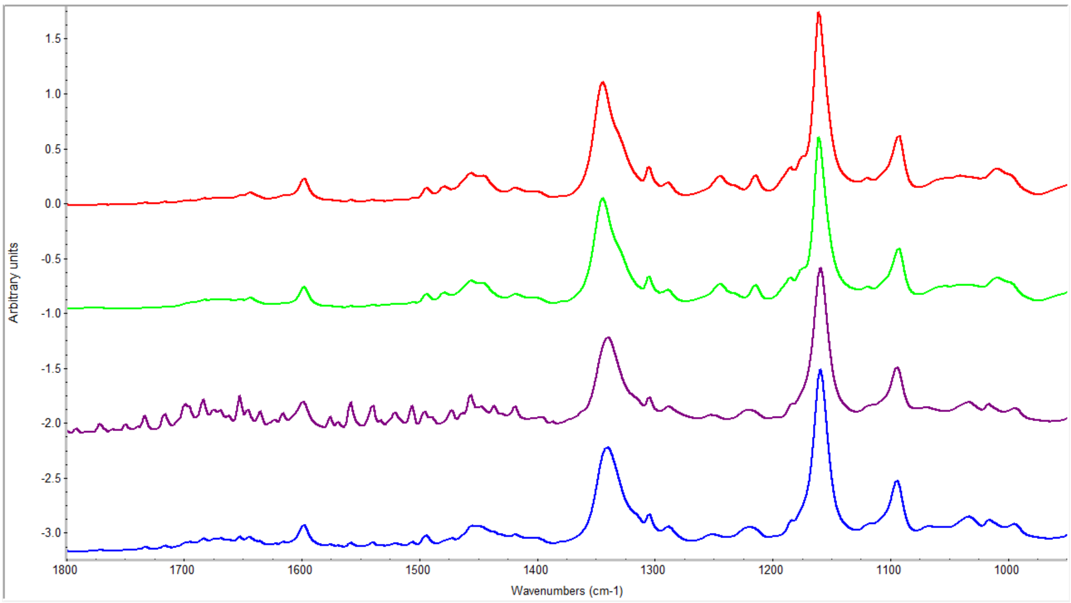

Figure S22. Experimental infra-red spectra for P1(blue), P2 (purple), P3 (green) and P4 (red) after subtraction of the spectrum for CDCl3 solvent alone.

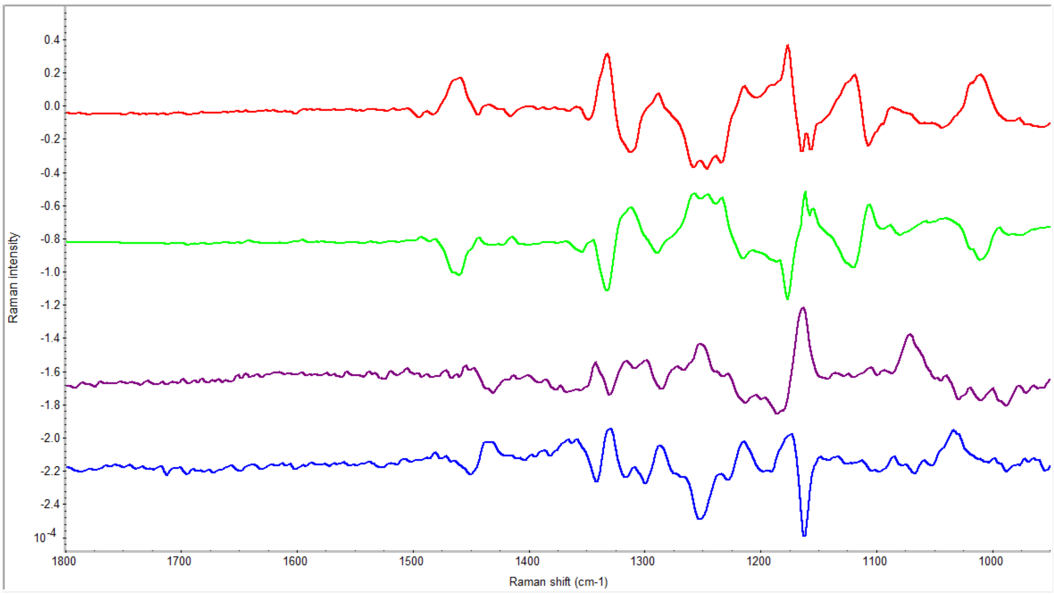

Figure S23. Experimental VCD spectra for P1(blue), P2 (purple), P3 (green) and P4 (red) after subtraction of the spectrum for CDCl3 solvent alone.

15.2. Computational Spectral Simulations

A Monte Carlo molecular mechanics search for low energy geometries was conducted for the RRR and RRS diastereomers. MacroModel within the Maestro graph-ical interface (Schrödinger Inc.) was used to generate 163 (RRR) and 134 (RRS) starting coordinates for conformers within 21 $kJmol^{-1}$ of the lowest energy conformer. These were used as starting points for density functional theory (DFT) minimizations within Gaussian16. REF Optimized structures, harmonic vibrational frequencies /intensities, VCD rotational strengths, and free energies at STP (including zero-point energies) were determined at the B3PW91/cc-pVTZ level of theory using the default polarizable continuum model (PCM) for chloroform.

<u>RRR diastereomer</u>

Thirty conformations were found within 5 kJmol-1 of the minimum and these are shown overlaid in Figure S24. The coordinates of the minimum energy conformation are shown in Table S7.

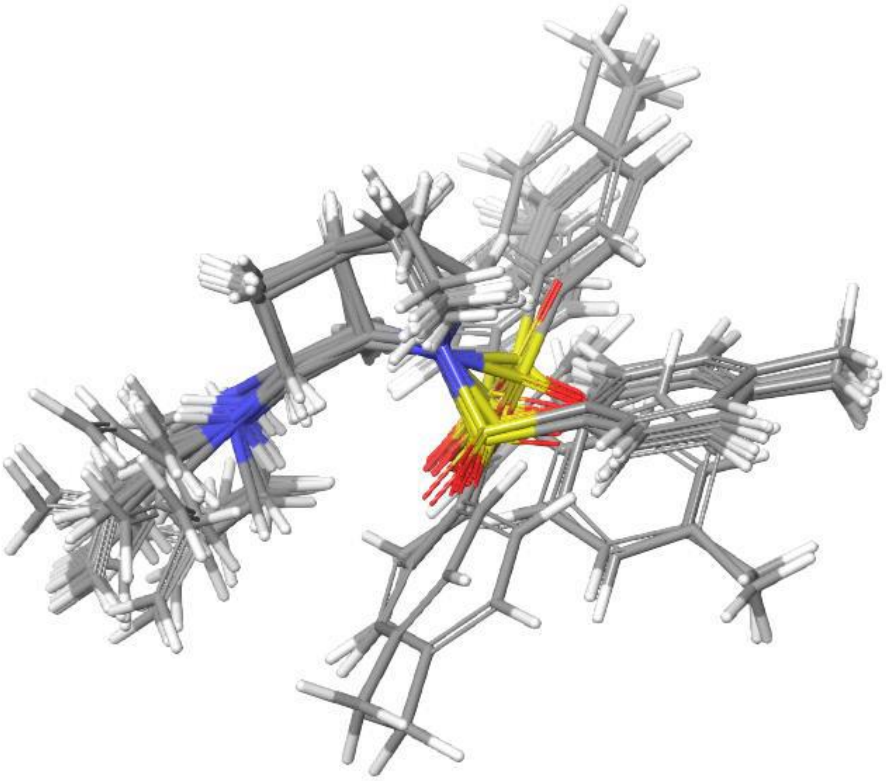

Figure S24. Overlay of the thirty lowest energy conformations (within 5 kJmol-1 of the minimum) used in the calculation of the Boltzmann average IR and VCD spectra for the RRR diastereomer.

Table S7. Coordinates for the minimum energy conformation of the *RRR* diastereomer.

C	0.21320	1.94700	0.81530
C	-0.45160	3.06700	0.01960
C	-1.63120	2.39450	-0.68130
C	-1.29920	0.87910	-0.70800
N	0.01120	0.78700	-0.06360
H	1.27670	2.11430	0.98670
H	-0.27060	1.79980	1.78640
H	0.24960	3.45180	-0.72300
H	-0.75520	3.89710	0.65860
C	-2.89130	2.09320	0.16570
H	-1.83990	2.84340	-1.65010
C	-2.55330	0.58260	0.15640
H	-1.29490	0.37090	-1.66940
H	-2.29340	0.19640	1.15280
N	-3.51430	-0.25040	-0.53510
H	-3.82510	2.32210	-0.34670
H	-2.90470	2.56080	1.15110
H	-3.07000	-1.13280	-0.76690
C	-4.71580	-0.51660	0.24820
C	-5.70450	-1.31190	-0.54190
H	-4.50160	-1.02580	1.20090
H	-5.16080	0.45220	0.50390
C	-6.23320	-2.46320	-0.14840
H	-6.95470	-2.99440	-0.75740
H	-5.96500	-2.91360	0.80170
H	-5.98100	-0.88940	-1.50450
S	0.57280	-0.69100	0.42190
O	0.38420	-0.89200	1.85210
O	-0.00190	-1.65770	-0.50100
C	5.06170	-0.43280	-0.34730
C	4.56630	-0.50350	0.95190
C	3.20110	-0.57210	1.19800
C	2.32110	-0.56690	0.12800
C	2.78800	-0.49750	-1.18080
C	4.14970	-0.43080	-1.40790
C	6.53610	-0.37490	-0.61400
H	5.25600	-0.50680	1.78760
H	2.81920	-0.63460	2.20790
H	2.09100	-0.50120	-2.00870
H	4.51620	-0.37770	-2.42650
H	6.78000	0.43610	-1.30280
H	7.10270	-0.22720	0.30460
H	6.88210	-1.30290	-1.07640

RRS Diastereomer

Fourteen conformations were found within 5 kJmol^{-1} of the minimum and these are shown overlaid in Figure S25. The coordinates of the minimum energy conformation are shown in Table S8.

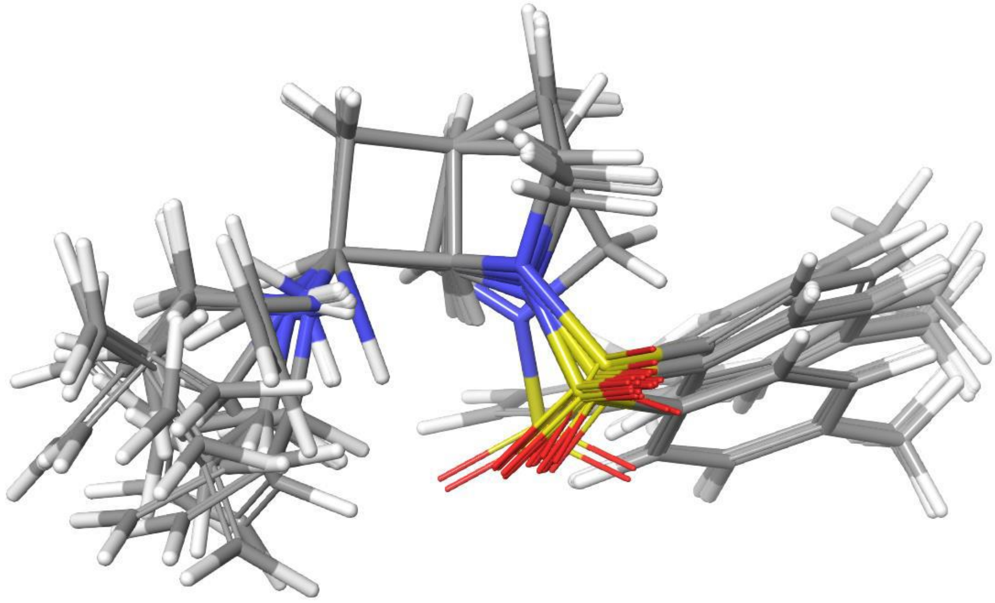

Figure S25. Overlay of the fourteen lowest energy conformations (within 5 kJmol-1 of the minimum) used in the calculation of the Boltzmann average IR and VCD spectra for the RRS diastereomer.

Table S8. Coordinates for the minimum energy conformation of the *RRS* diastereomer.

C	-0.32790	1.54360	1.56760
C	-0.22740	2.83370	0.75280
C	-0.93790	2.51260	-0.55470
C	-0.81390	0.97910	-0.75150
N	-0.37310	0.48980	0.54680
H	0.51390	1.39650	2.24240
H	-1.24220	1.51260	2.16530
H	0.82120	3.06730	0.55650
H	-0.66910	3.68180	1.27780
C	-2.48000	2.36770	-0.52950
H	-0.57120	3.10290	-1.39210
C	-2.34110	0.90560	-1.01760
H	-0.17700	0.60100	-1.55010
N	-3.07120	-0.10470	-0.29660
H	-3.03670	3.04910	-1.17120
H	-2.89080	2.41550	0.48050
S	0.38980	-0.94490	0.71100
O	0.40080	-1.26000	2.12770
O	-0.22330	-1.85050	-0.24750
C	4.71470	-0.26950	-0.60690
C	4.33160	-0.07660	0.72100
C	3.02700	-0.29730	1.13200
C	2.08110	-0.71470	0.20270
C	2.43890	-0.92220	-1.12410
C	3.74860	-0.69600	-1.51800
C	6.13670	-0.05930	-1.03500
H	5.06850	0.24530	1.44770
H	2.74710	-0.16590	2.16860
H	1.70280	-1.27590	-1.83320
H	4.02630	-0.86140	-2.55250
H	6.62500	0.70630	-0.43160

H	6.71310	-0.98170	-0.92020
H	6.19770	0.23440	-2.08310
C	-4.46850	-0.20550	-0.69270
H	-4.59880	-0.44660	-1.76040
C	-5.19380	-1.21000	0.14380
C	-5.89490	-2.22760	-0.33820
H	-2.49980	0.85420	-2.10650
H	-4.92450	0.78020	-0.53870
H	-2.60810	-1.00070	-0.40550
H	-5.11350	-1.06770	1.21840
H	-6.41110	-2.92140	0.31440
H	-5.97990	-2.40120	-1.40610

15.3. Fit between calculated and experimental IR and VCD spectra

An in-house program was used to generate Boltzmann weighted average spectra for the conformations with the 5 $kJmol^{-1}$ limit and to fit Lorentzian line shapes (12 cm^{-1} line width) to the computed spectra applying a linear scaling factor of 0.98. The fits between calculated and experimental IR and VCD data are shown in Figures S5 (IR) and S6 and S7 (VCD). There is reasonable agreement between calculated and experimental IR spectra. The major differences are in the intense peaks calculated at 1135 and 1310 cm^{-1} but observed at 1160 and 1340 cm^{-1}. The agreement between calculated and experimental VCD spectra is better with agreement in the signs and positions of peaks across most of the spectrum. There is a better match visually with P2 (green) and the calculated RRR diastereomer (red) as shown in Figure S28, and with P3 (blue) and the calculated RRS diastereomer (red) as shown in Figure S27.

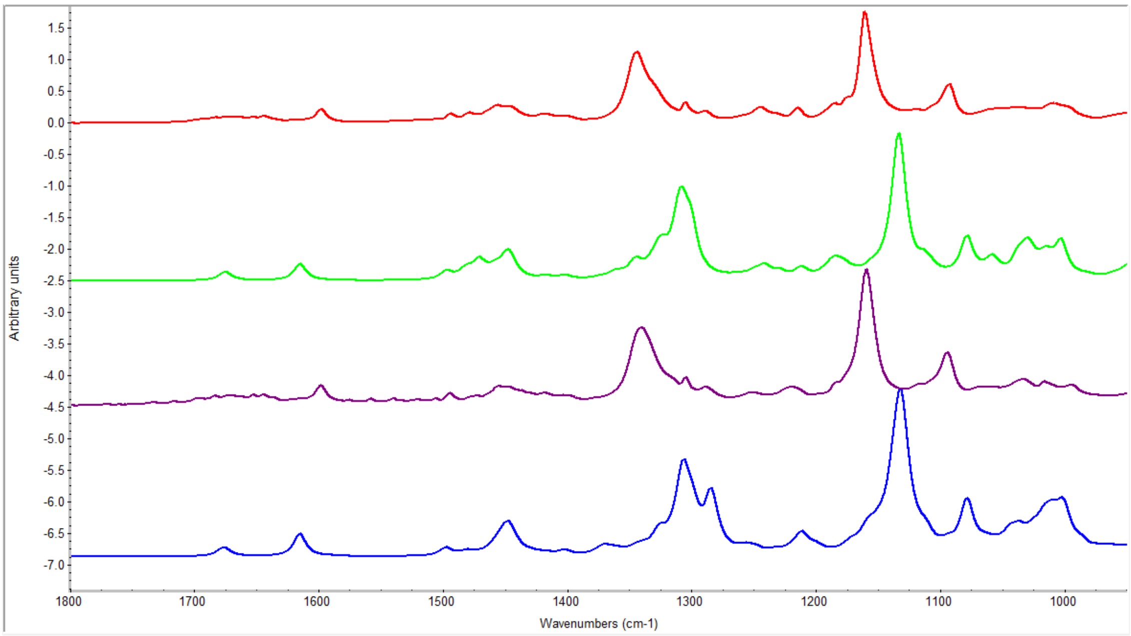

Figure S26. Comparison of experimental and calculated infra-red spectra. The figure shows experimental data for P1 (purple) and P3 (red) and calculated data for RRR (blue) and RRS (green)

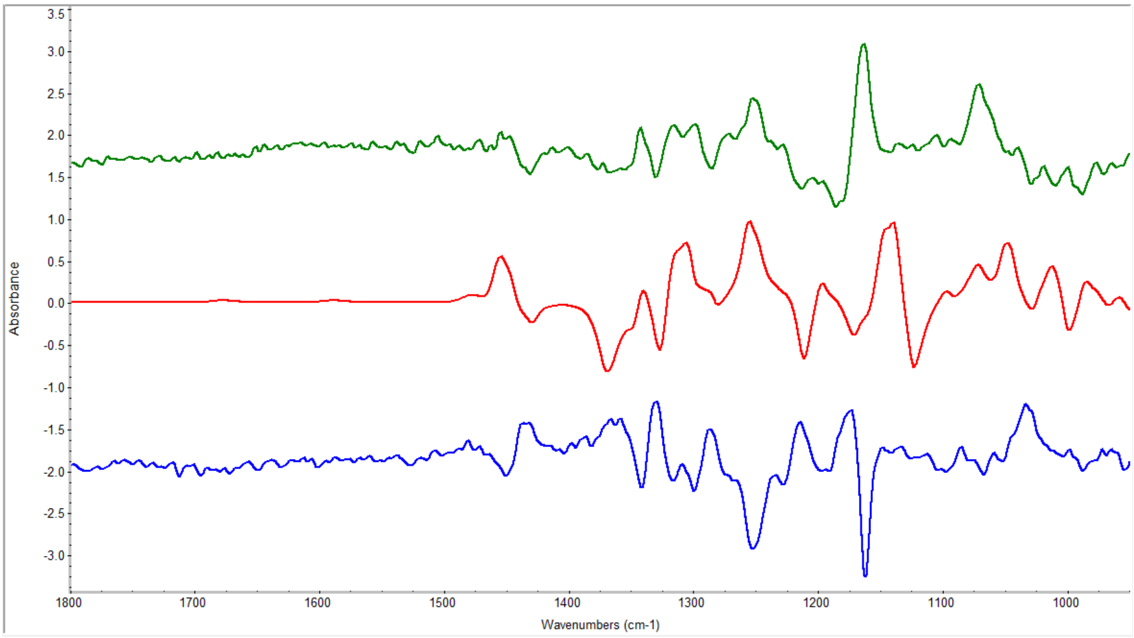

Figure S27. Comparison of experimental and calculated VCD spectra for the RRR diastereomer. The figure shows experimental data for P1 (blue) and P2 (green) and calculated data (red).

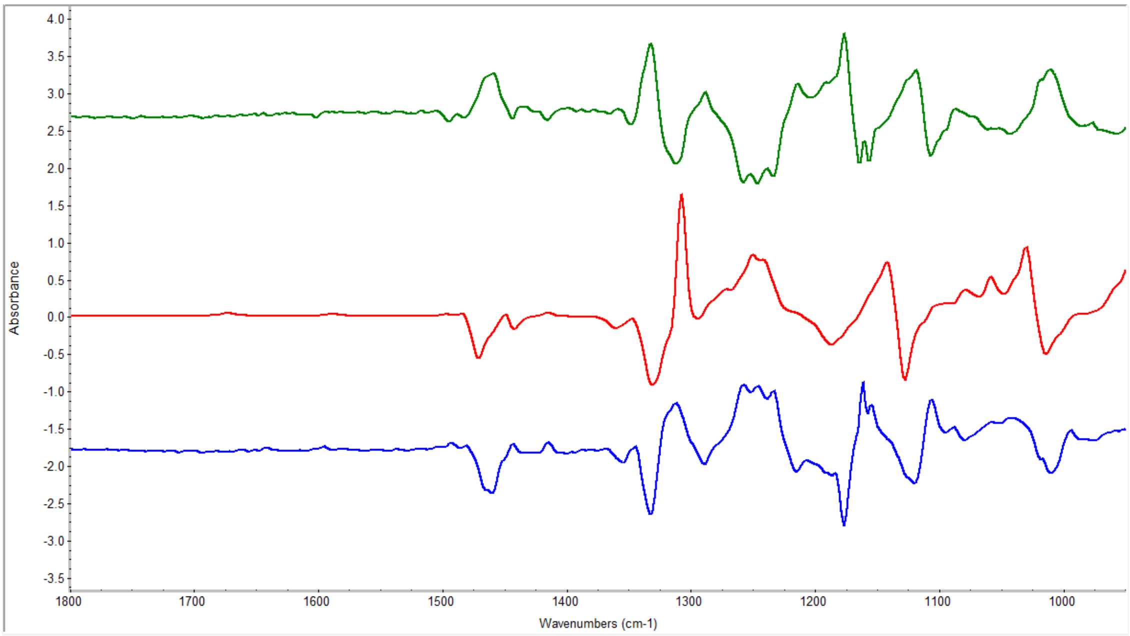

Figure S28. Comparison of experimental and calculated VCD spectra for the RRS diastereomer. The figure shows experimental data for P3 (blue) and P4 (green) and calculated data (red).

<u>Automatic fitting of data using the Cai factor</u>

The data was also analyzed automatically using the Cai algorithm (REF In Press). The Cai factor obtained considering P1 & P2 together with the calculated RRR data is 38 (fairly confident) in favour of P2 matching the RRR diastereomer. Considering P3 & P4 together with calculated RRS data gives 57 (very confident) in favour of P3 matching the RRS diastereomer. This is in agreement with the visual analysis described above.

If we instead compare P1 & P2 with calculated RRS data and P3 & P4 with calculated RRR data we obtain somewhat lower Cai factors of 27 and 43 indicating the support for the assignment of P1/P2 as SSS/RRR and P3/P4 as RRS/SSR.

Summary Result: The VCD results suggest the following stereochemical assignments with reasonable certainty.

P1 = SSS

P2 = RRR

P3 = RRS

P4 = SSR

Considering VCD Cai factor for P3 matching the RRS diastereomer (57), P2 matching the RRR diastereomer (38) and how area between peaks varies across different variants, we can assign absolute configuration with very high certainty.

16. NMR analysis

16.1. Bicyclic ketone synthesis

16.1.1. *N*-(But-3-en-1-yl)-4-methylbenzenesulfonamide

^{1}H NMR (400 MHz, $CDCl_3$)

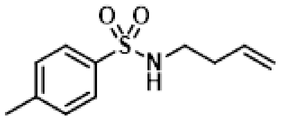

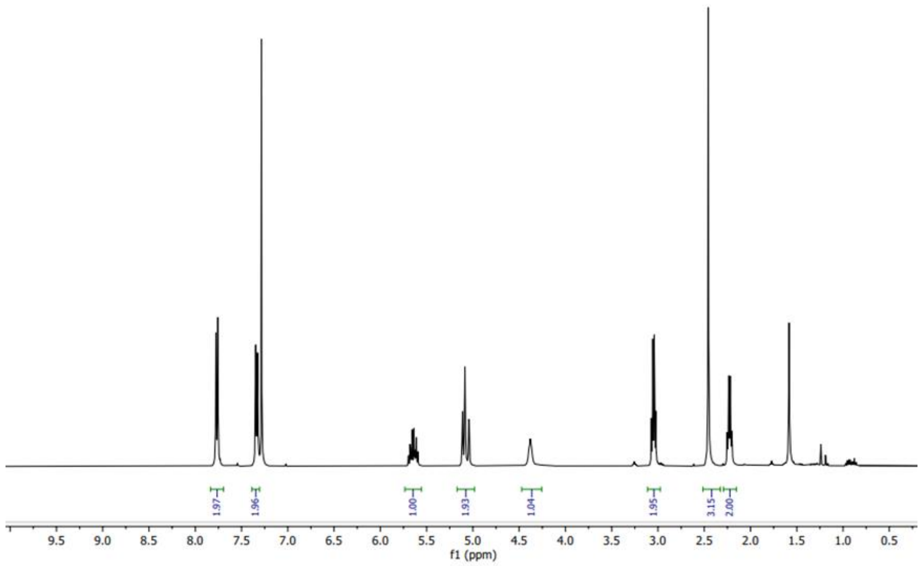

^{13}C NMR (101 MHz, CDCl$_3$)

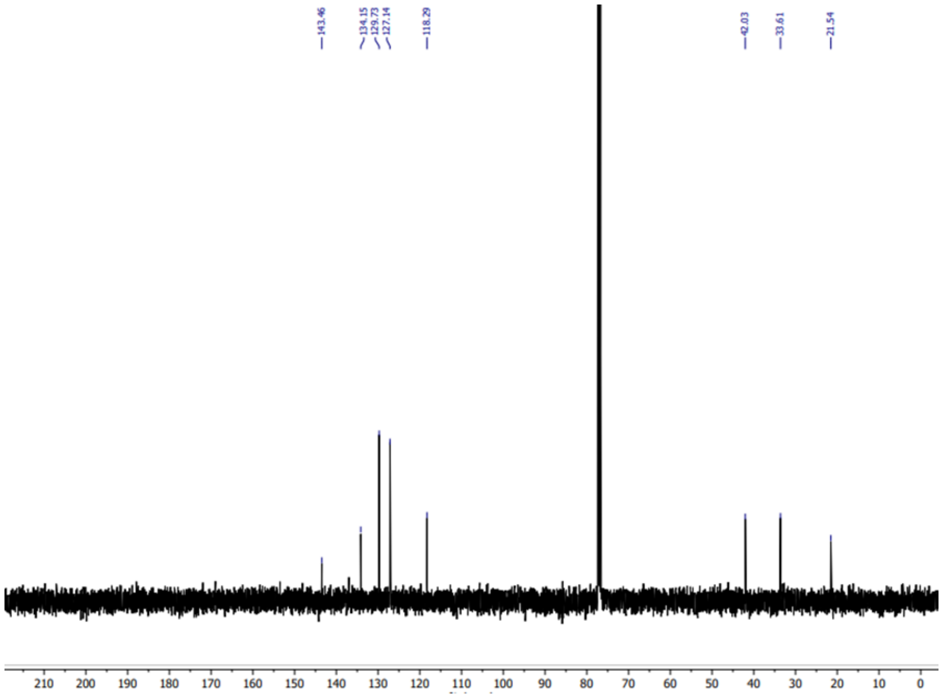

16.1.2. *N*-(But-3-en-1-yl)-4-methyl-*N*-(2-oxo-2-(pyrrolidin-1-yl)ethyl)benzenesulfonamide

^{1}H NMR (400 MHz, CDCl$_3$)

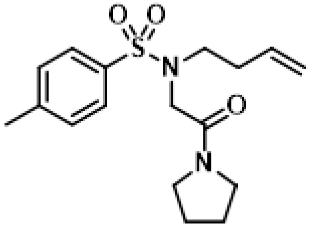

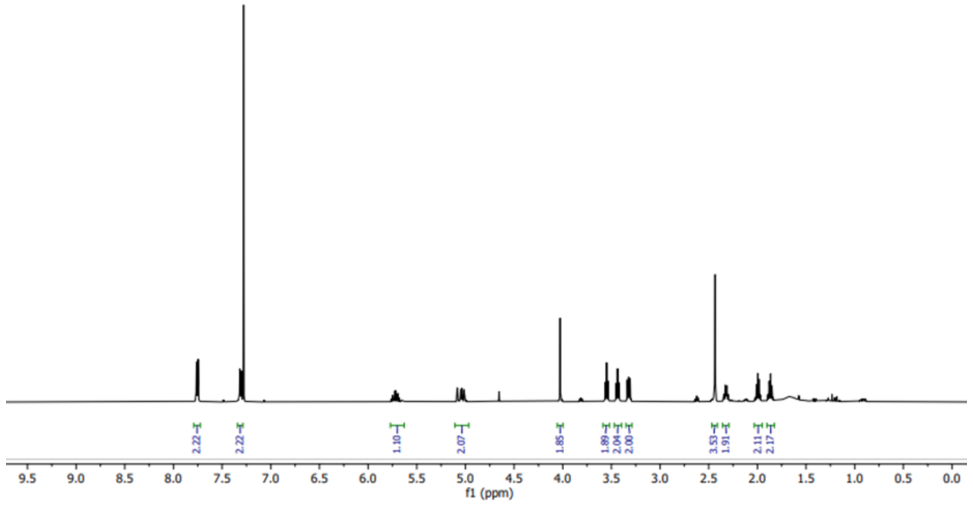

^{13}C NMR (101 MHz, $CDCl_3$)

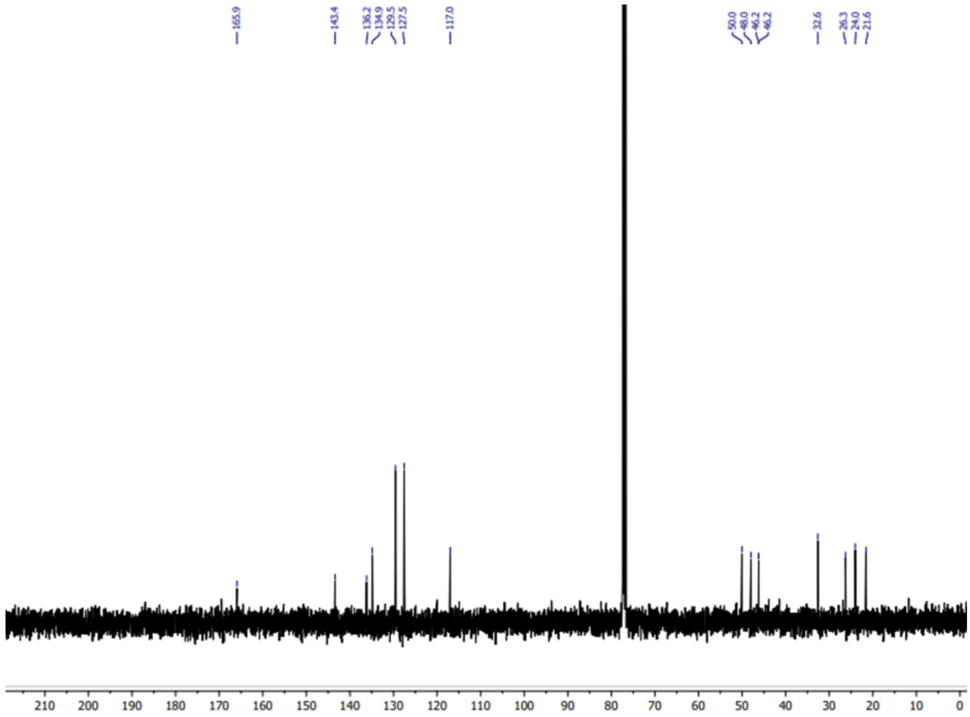

16.1.3. *rac*-(1*S*,5*S*)-2-Tosyl-2-azabicyclo[3.2.0]heptan-7-one (2)

^{1}H NMR (400 MHz, $CDCl_3$)

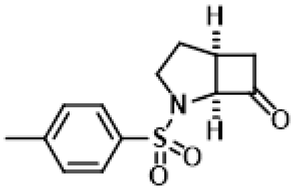

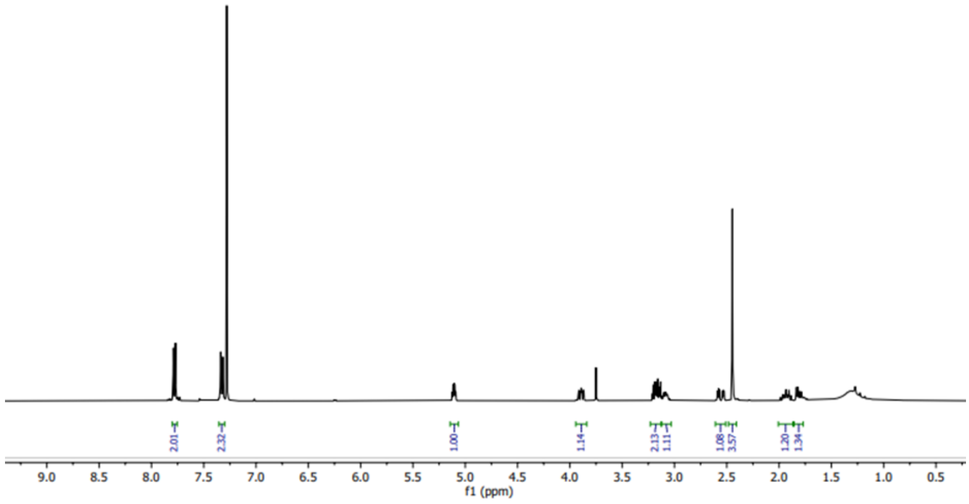

^{13}C NMR (101 MHz, CDCl$_3$)

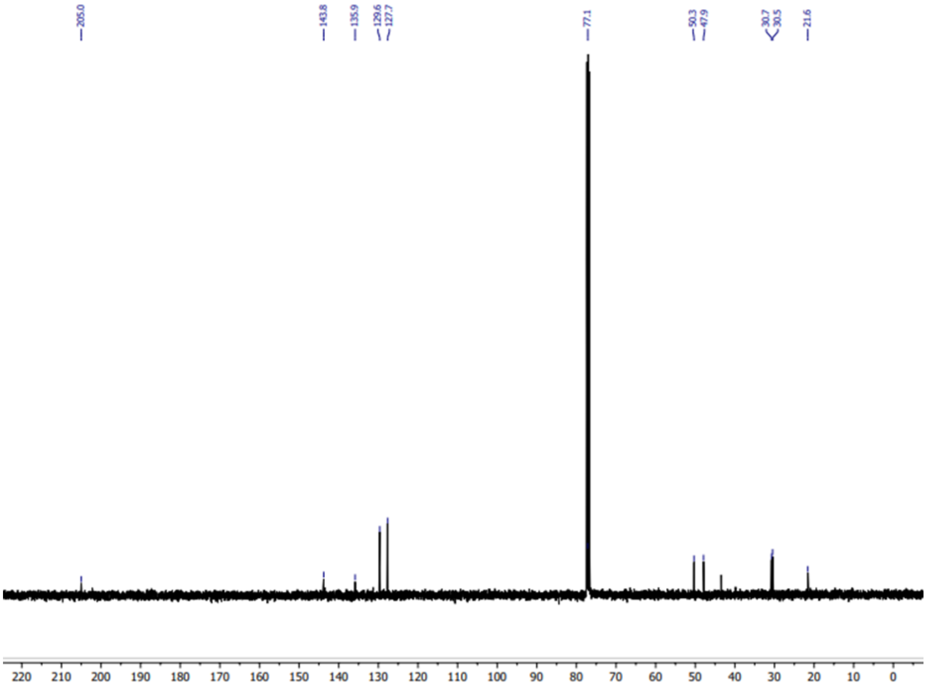

16.2. Isolated products spectra

16.2.1. (*S*,*S*,*S*)-3

^{1}H NMR (500 MHz, CDCl$_3$) δ 1.57 – 1.68 (m, 2H), 1.69 – 1.85 (m, 2H), 2.41 (s, 4H), 2.81 – 2.91 (m, 1H), 3.12 – 3.28 (m, 3H), 3.42 – 3.50 (m, 1H), 3.51 – 3.59 (m, 1H), 3.97 (dd, *J* = 7.6, 2.9 Hz, 1H), 5.08 (d, *J* = 10.2 Hz, 1H), 5.18 (dd, *J* = 17.2, 1.9 Hz, 1H), 5.87 (ddt, *J* = 16.5, 10.3, 6.0 Hz, 1H), 7.25 – 7.34 (m, 3H), 7.70 (d, *J* = 8.0 Hz, 2H); ^{13}C NMR (126 MHz, CDCl$_3$) δ 21.7, 30.0, 30.9, 34.7, 49.2, 49.5, 58.4, 64.8, 116.3, 127.3, 129.8, 136.2, 136.4, 143.5 ppm.

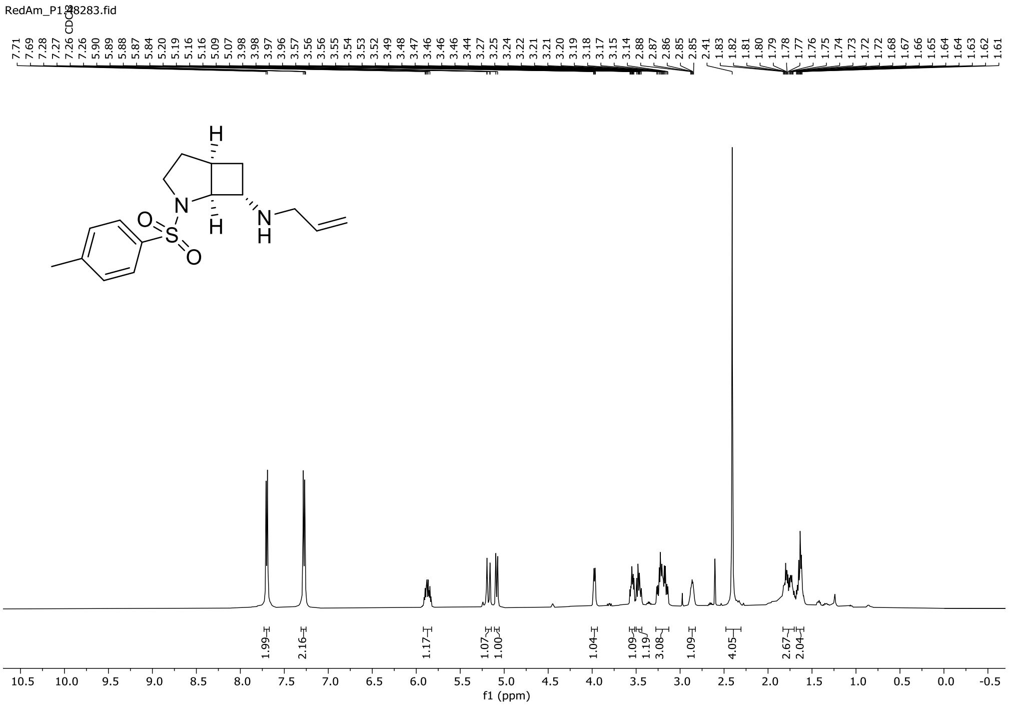

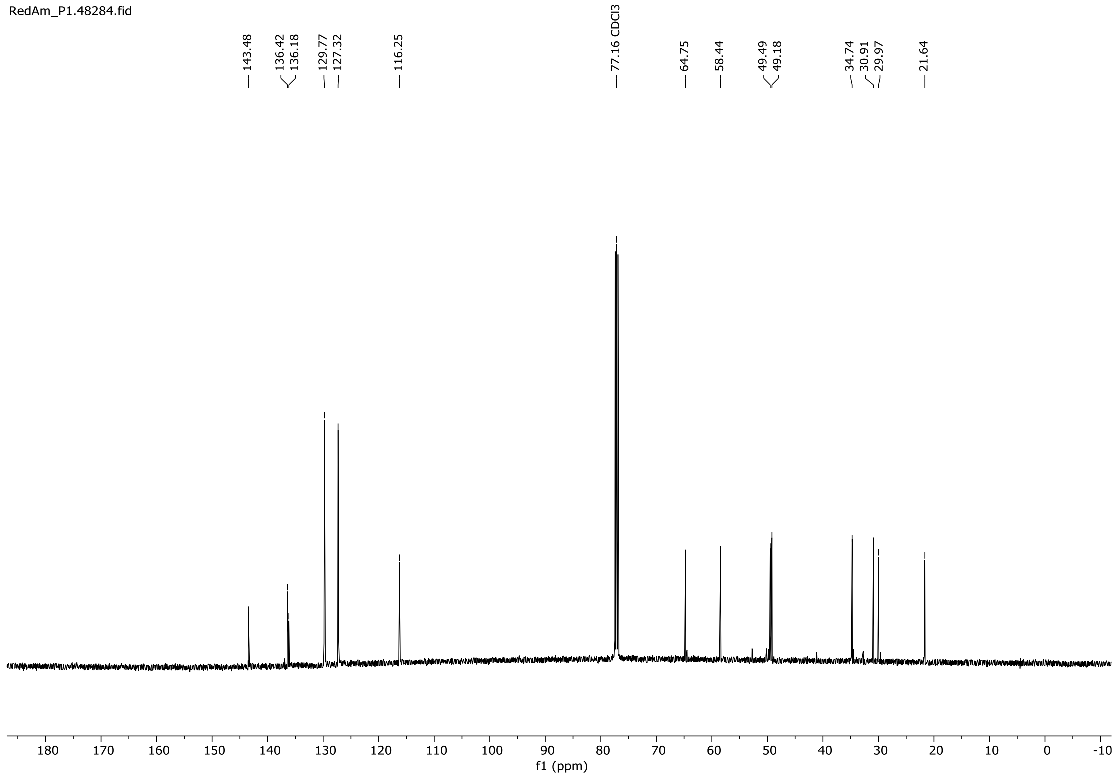

16.2.2. (*R*,*R*,*R*)-3

^{1}H NMR (600 MHz, $CDCl_3$) δ 1.60 – 1.85 (m, 5H), 2.42 (s, 3H), 2.88 (s, 1H), 3.21 (d, *J* = 31.5 Hz, 3H), 3.44 – 3.51 (m, 1H), 3.55 (dt, *J* = 7.6, 4.0 Hz, 1H), 3.98 (d, *J* = 7.3 Hz, 1H), 5.10 (d, *J* = 10.2 Hz, 1H), 5.19 (d, *J* = 17.0 Hz, 1H), 5.84 – 5.94 (m, 1H), 7.29 (d, *J* = 7.8 Hz, 2H), 7.71 (d, *J* = 7.9 Hz, 2H); ^{13}C NMR (151 MHz, $CDCl_3$) δ 21.7, 29.8, 31.0, 34.8, 49.2, 49.5, 58.5, 64.8, 116.3, 127.4, 129.8, 136.3, 136.5, 143.5.

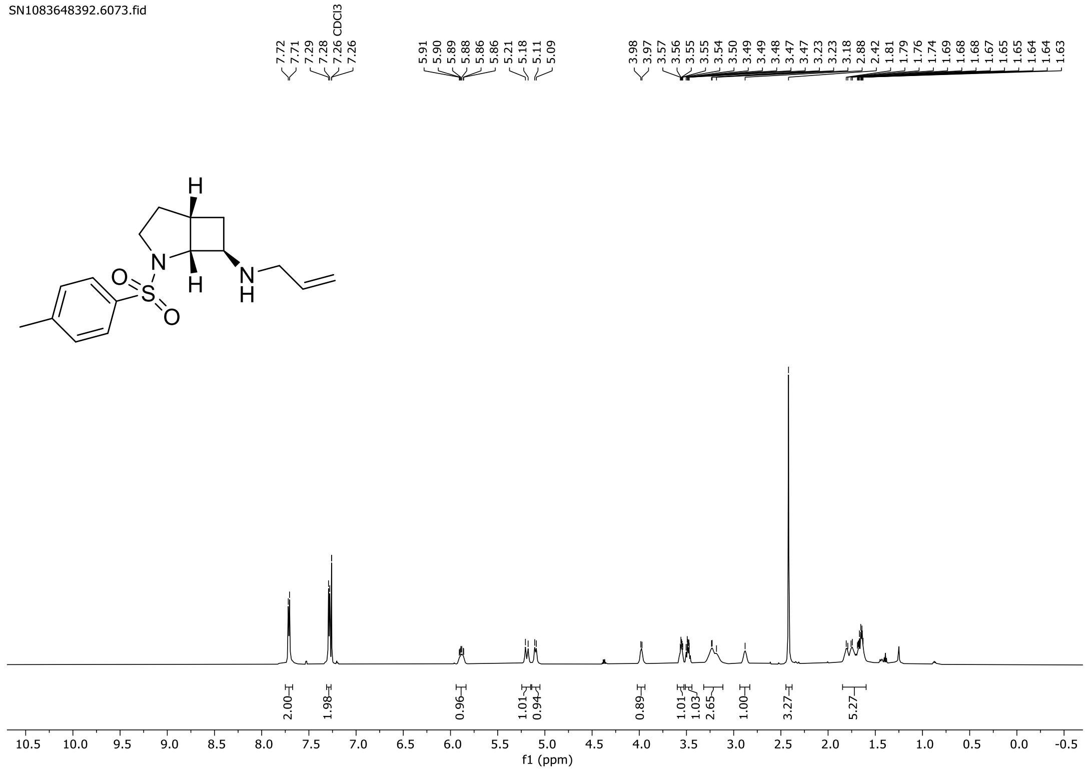

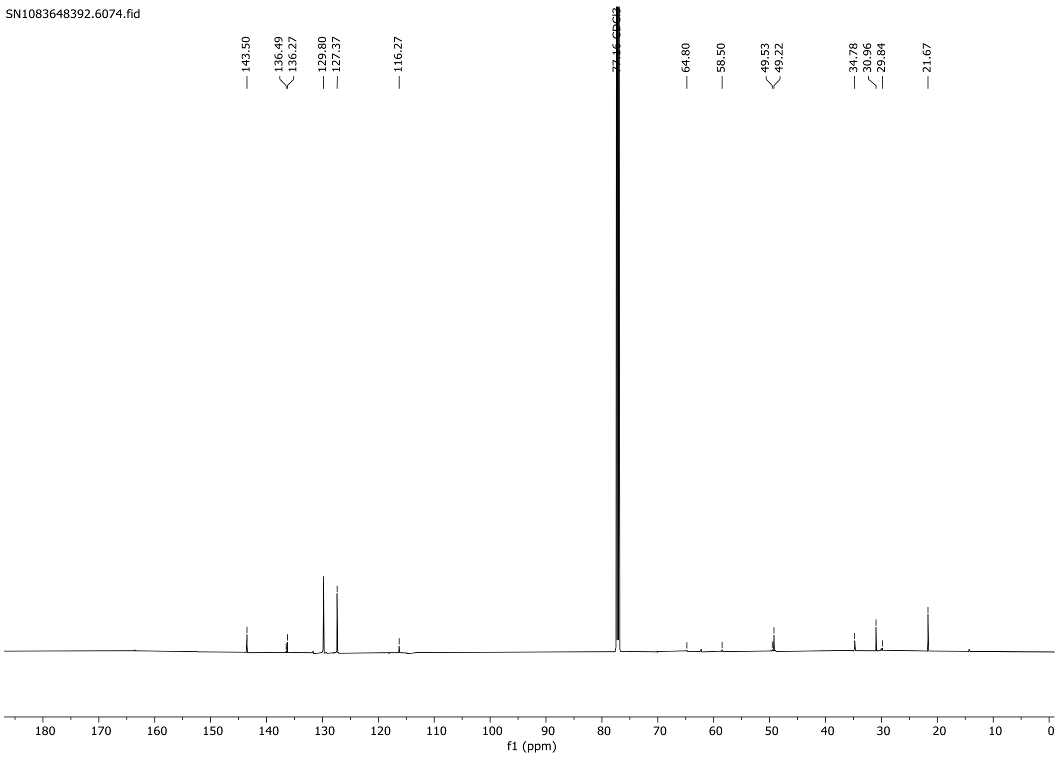

16.2.3. (*S*,*S*,*R*)-3

^{1}H NMR (500 MHz, $CDCl_3$) δ 1.02 – 1.13 (m, 1H), 1.30 – 1.39 (m, 1H), 1.39 – 1.47 (m, 1H), 1.83 (s, 1H), 2.30 – 2.40 (m, 1H), 2.41 (s, 3H), 2.67 (p, *J* = 7.2 Hz, 1H), 3.14 – 3.22 (m, 1H), 3.36 (td, *J* = 9.0, 5.8 Hz, 1H), 3.42 – 3.55 (m, 2H), 3.81 (dd, *J* = 12.3, 8.4 Hz, 1H), 4.46 (td, *J* = 5.9, 2.8 Hz, 1H), 5.09 (dq, *J* = 10.2, 1.5 Hz, 1H), 5.23 (dq, *J* = 17.2, 1.7 Hz, 1H), 5.85 – 5.98 (m, 1H), 7.28 (d, *J* = 8.0 Hz, 2H), 7.70 (d, *J* = 8.3 Hz, 2H); ^{13}C NMR (126 MHz, $CDCl_3$) δ 21.7, 29.7, 32.8, 34.5, 49.9, 50.2, 52.8, 64.5, 116.2, 127.3, 129.9, 136.2, 137.0, 143.5 ppm.

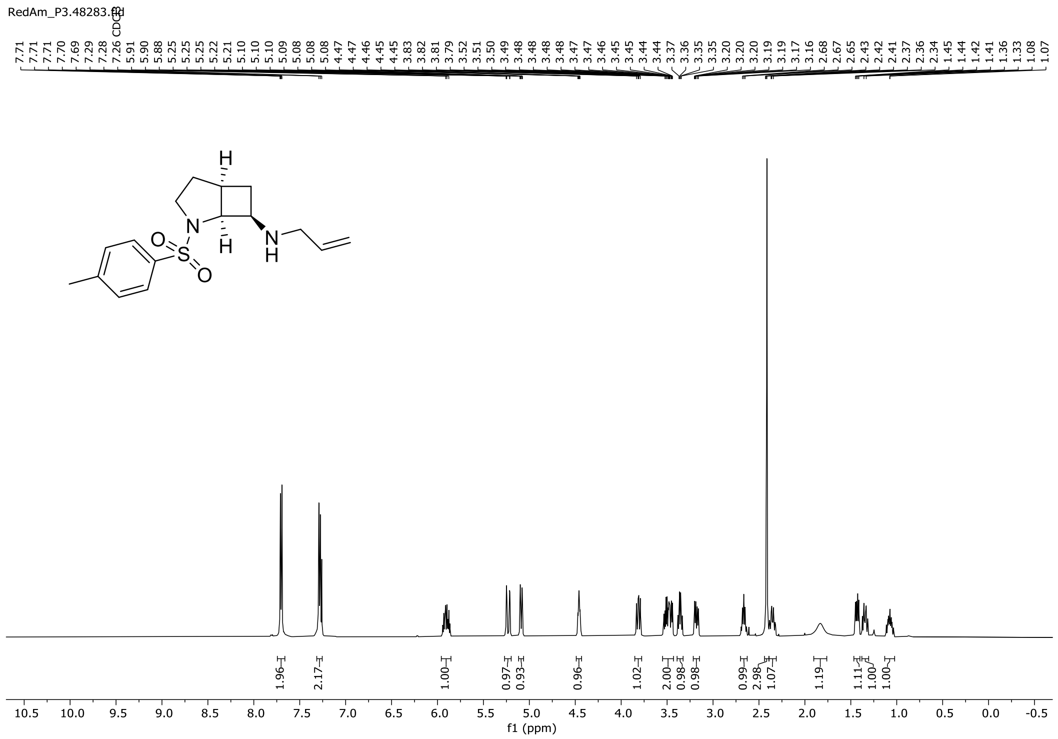

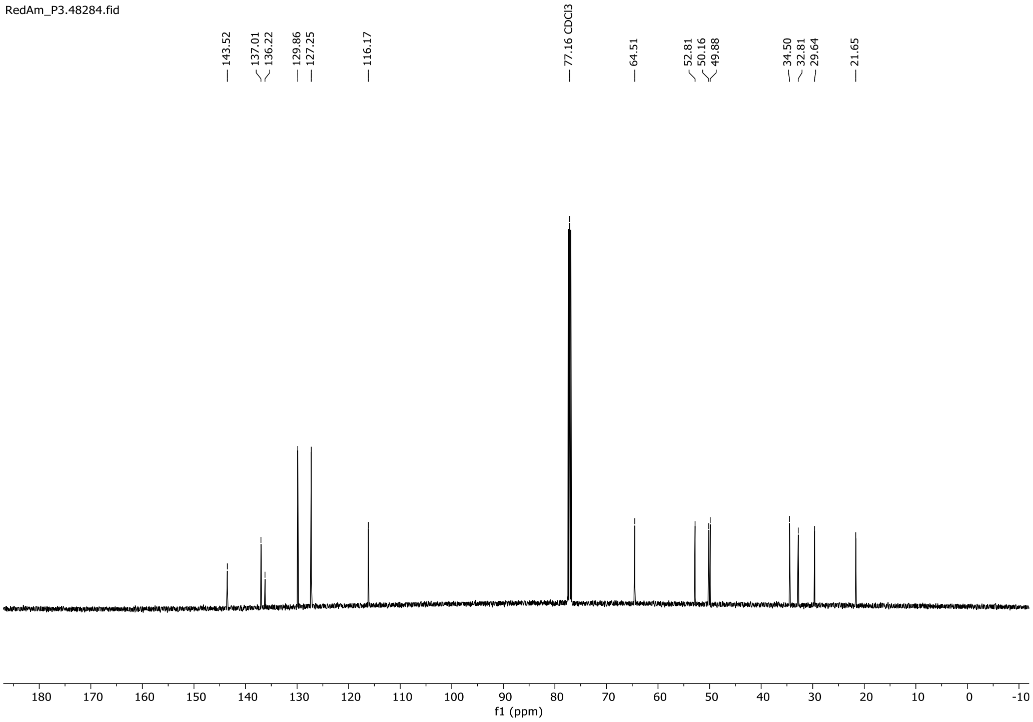

16.2.4. (*R*,*R*,*S*)-3

^{1}H NMR (500 MHz, $CDCl_3$) δ 1.03 – 1.13 (m, 1H), 1.31 – 1.39 (m, 1H), 1.43 (dd, *J* = 12.6, 5.9 Hz, 1H), 1.94 – 2.09 (m, 1H), 2.32 – 2.38 (m, 1H), 2.41 (s, 3H), 2.63 – 2.70 (m, 1H), 3.15 – 3.22 (m, 1H), 3.33 – 3.39 (m, 1H), 3.44 – 3.54 (m, 2H), 3.81 (dd, *J* = 12.3, 8.4 Hz, 1H), 4.42 – 4.49 (m, 1H), 5.09 (d, *J* = 10.2 Hz, 1H), 5.23 (d, *J* = 17.1 Hz, 1H), 5.84 – 5.96 (m, 1H), 7.28 (d, *J* = 8.0 Hz, 2H), 7.70 (d, *J* = 8.0 Hz, 2H); ^{13}C NMR (126 MHz, $CDCl_3$) δ 21.6, 29.6, 32.7, 34.5, 49.8, 50.2, 64.5, 116.2, 127.2, 129.9, 136.2, 136.9, 143.5.

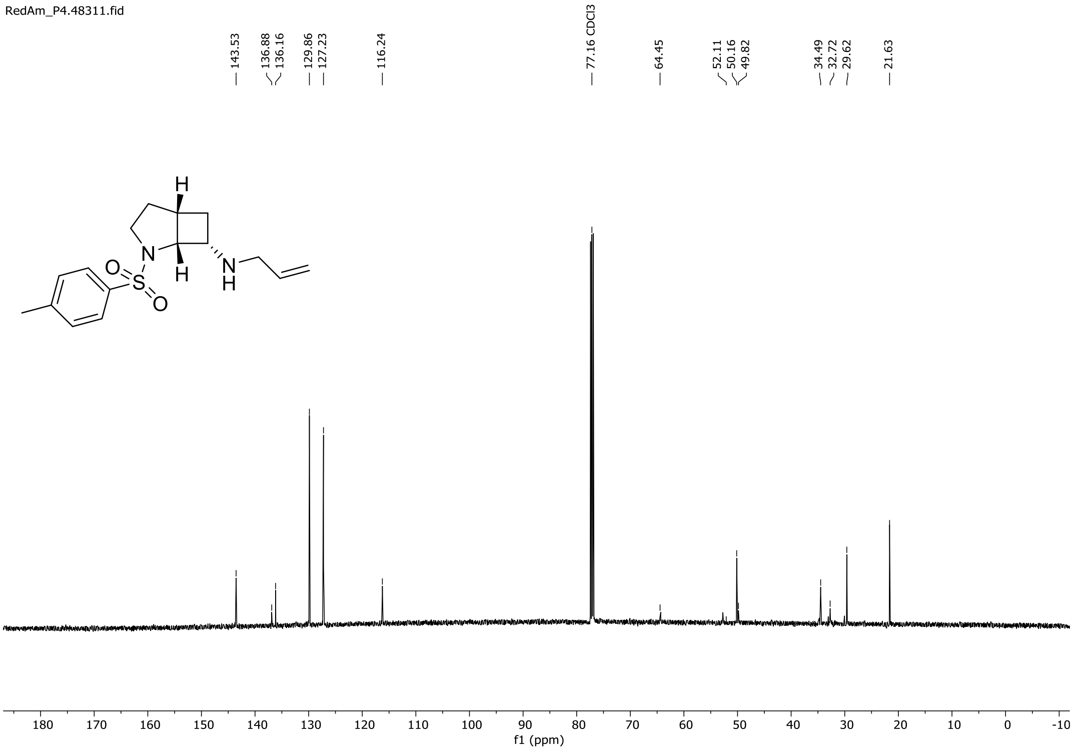

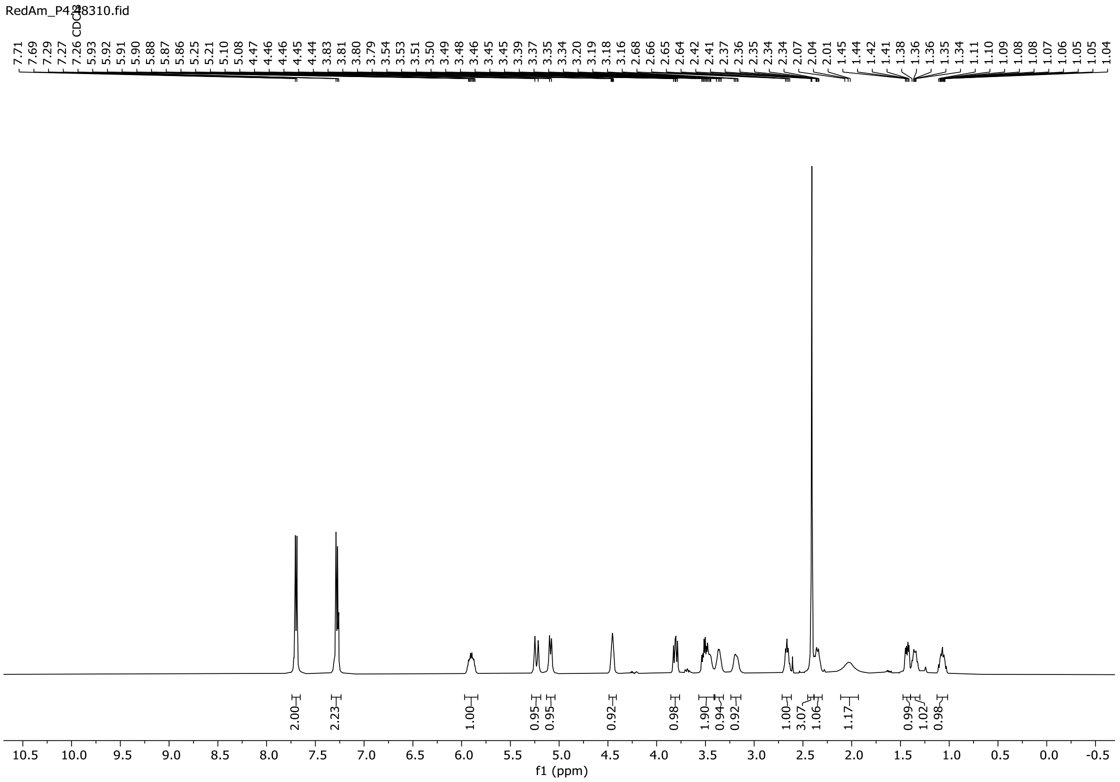

16.2.5. Deallylated 3 product

(1S,5S,7S)-2-Tosyl-2-azabicyclo[3.2.0]heptan-7-amine

^{1}H NMR (400 MHz, $CDCl_3$)

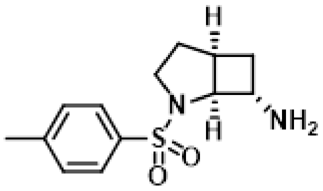

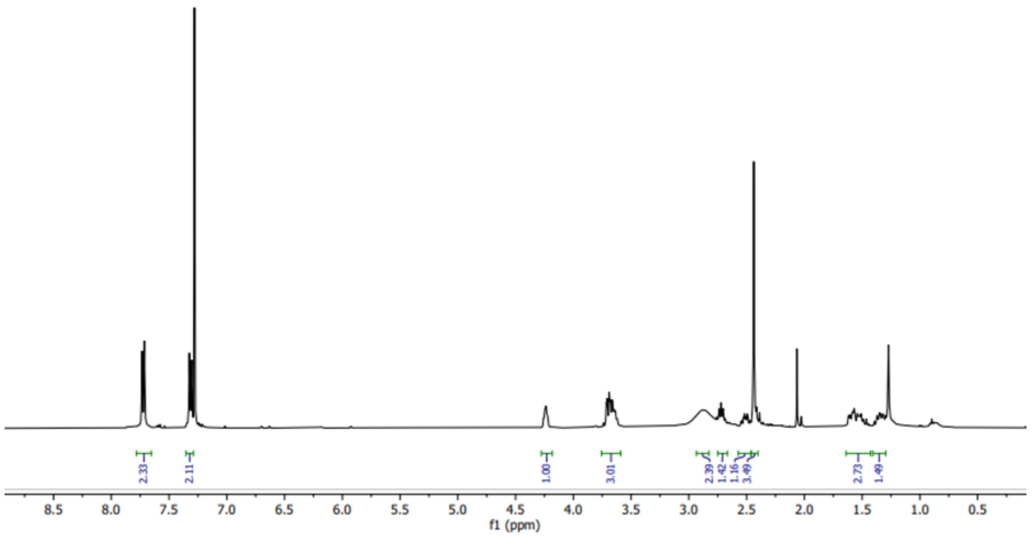

^{13}C NMR (101 MHz, $CDCl_3$)

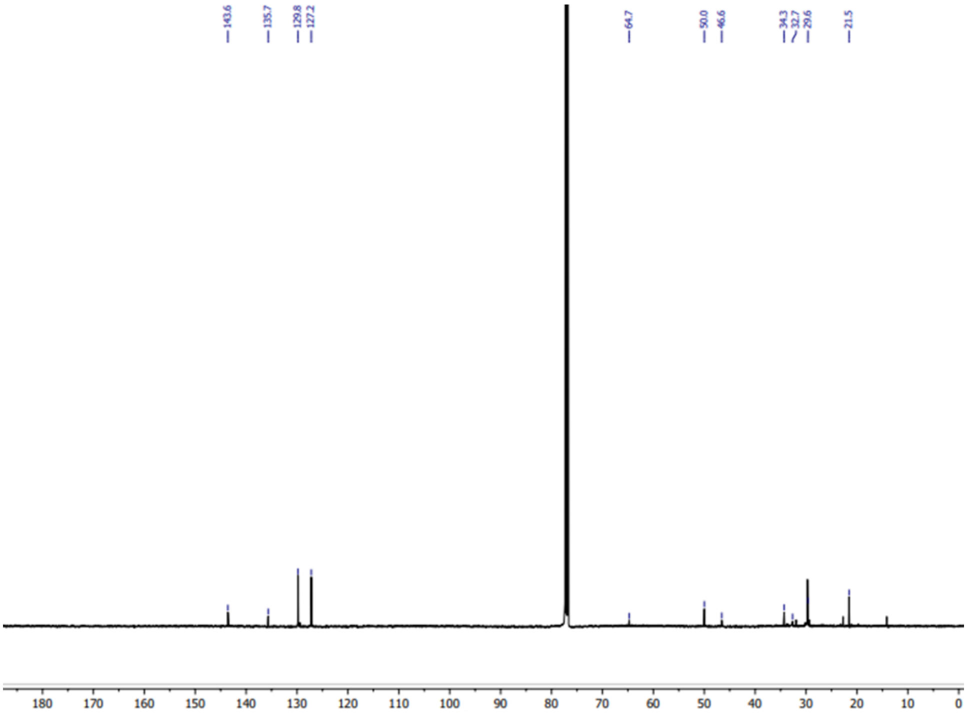

17.References

Bicyclic ketone synthesis

D. Belmessiere, D. B. Cordes, A. M. Z. Slawin, A. D. Smith, *Org. Lett.* 2013, 15, 13, 3472–3475.

VCD references

Gaussian 16, Revision B.01, M. J. Frisch, G. W. Trucks, H. B. Schlegel, G. E. Scuseria, M. A. Robb, J. R. Cheeseman, G. Scalmani, V. Barone, G. A. Petersson, H. Nakatsuji, X. Li, M. Caricato, A. V. Marenich, J. Bloino, B. G. Janesko, R. Gomperts, B. Mennucci, H. P. Hratchian, J. V. Ortiz, A. F. Izmay-lov, J. L. Sonnenberg, D. Williams-Young, F. Ding, F. Lipparini, F. Egidi, J. Goings, B. Peng, A. Petrone,

T. Henderson, D. Ranasinghe, V. G. Zakrzewski, J. Gao, N. Rega, G. Zheng, W. Liang, M. Hada, M. Ehara, K. Toyota, R. Fukuda, J. Hasegawa, M. Ishida, T. Nakajima, Y. Honda, O. Kitao, H. Nakai,T. Vreven, K. Throssell, J. A. Montgomery, Jr., J. E. Peralta, F. Ogliaro, M. J. Bearpark, J. J. Heyd, E. N. Brothers, K. N. Kudin, V. N. Staroverov, T. A. Keith, R. Kobayashi, J. Normand, K. Raghavachari, A. P. Rendell, J. C. Burant, S. S. Iyengar, J. Tomasi, M. Cossi, J. M. Millam, M. Klene, C. Adamo, R. Cammi, J. W. Ochterski, R. L. Martin, K. Morokuma, O. Farkas, J. B. Foresman, and D. J. Fox, Gaussian, Inc., Wallingford CT, 2016.

Interpreting Vibrational Circular Dichroism Spectra: the CaiFactor for Absolute Configuration with Confidence, J. Lam, R. J. Lewis, J. M. Goodman. *Journal of Cheminformatics* 2023 in press
